# Influence of Vitamin D Status and Vitamin D_3_ Supplementation on Genome Wide Expression of White Blood Cells: A Randomized Double-Blind Clinical Trial

**DOI:** 10.1371/journal.pone.0058725

**Published:** 2013-03-20

**Authors:** Arash Hossein-nezhad, Avrum Spira, Michael F. Holick

**Affiliations:** 1 Department of Medicine, Section of Endocrinology, Nutrition, and Diabetes, Vitamin D, Skin and Bone Research Laboratory, Boston University Medical Center, Boston, Massachusetts, United States of America; 2 Department of Medicine, Section of Computational Biomedicine, Boston University Medical Center, Boston, Massachusetts, United States of America; Roswell Park Cancer Institute, United States of America

## Abstract

**Background:**

Although there have been numerous observations of vitamin D deficiency and its links to chronic diseases, no studies have reported on how vitamin D status and vitamin D_3_ supplementation affects broad gene expression in humans. The objective of this study was to determine the effect of vitamin D status and subsequent vitamin D supplementation on broad gene expression in healthy adults. (Trial registration: ClinicalTrials.gov NCT01696409).

**Methods and Findings:**

A randomized, double-blind, single center pilot trial was conducted for comparing vitamin D supplementation with either 400 IUs (n = 3) or 2000 IUs (n = 5) vitamin D_3_ daily for 2 months on broad gene expression in the white blood cells collected from 8 healthy adults in the winter. Microarrays of the 16 buffy coats from eight subjects passed the quality control filters and normalized with the RMA method. Vitamin D_3_ supplementation that improved serum 25-hydroxyvitamin D concentrations was associated with at least a 1.5 fold alteration in the expression of 291 genes. There was a significant difference in the expression of 66 genes between subjects at baseline with vitamin D deficiency (25(OH)D<20 ng/ml) and subjects with a 25(OH)D>20 ng/ml. After vitamin D_3_ supplementation gene expression of these 66 genes was similar for both groups. Seventeen vitamin D-regulated genes with new candidate vitamin D response elements including TRIM27, CD83, COPB2, YRNA and CETN3 which have been shown to be important for transcriptional regulation, immune function, response to stress and DNA repair were identified.

**Conclusion/Significance:**

Our data suggest that any improvement in vitamin D status will significantly affect expression of genes that have a wide variety of biologic functions of more than 160 pathways linked to cancer, autoimmune disorders and cardiovascular disease with have been associated with vitamin D deficiency. This study reveals for the first time molecular finger prints that help explain the nonskeletal health benefits of vitamin D.

**Trial Registration:**

ClinicalTrials.gov NCT01696409

## Introduction

Vitamin D deficiency defined as a serum concentration of 25-hydroxyvitamin D [25(OH)D]<20 ng/ml is the largest pandemic in the world [1],[2]. The musculoskeletal consequences of inadequate vitamin D are well-established [2],[3]. In addition to its traditional role in calcium homeostasis, a growing number of other conditions have also been linked to vitamin D insufficiency including cancers, autoimmune diseases, infectious diseases, type 2 diabetes and cardiovascular disease [1],[2],[3],[4].

Epidemiological studies have suggested that adequate levels of 25(OH)D are critical for the prevention of various solid tumors, including breast, ovarian and colon cancers [4],[5]. The risk of developing and dying of these cancers appears to be inversely correlated with sun exposure, and/or vitamin D status, suggesting that vitamin D has chemopreventive properties [4],[5]. Studies based on immunomodulatory effects of vitamin D have recommended vitamin D supplementation for prevention of autoimmune diseases and several forms of cancer [4],[6],[7].

Vitamin D requires two metabolic conversions, 25-hydroxylation in the liver and 1α-hydroxylation in the kidney, before its hormonal form, 1,25-dihydroxyvitamin D[1,25(OH)_2_D], can bind to the vitamin D receptor (VDR) to modulate gene transcription. The VDR is present in a wide variety of cells and tissues and 1,25(OH)_2_D via its receptor directly or indirectly have been reported to have effects on cell cycling and proliferation, differentiation, apoptosis and the production of cathelicidin, renin and insulin [2],[3],[4].

The wide distribution of VDR and the plethora of biologic actions reported for 1,25(OH)_2_D may explain how vitamin D can prevent a variety of diseases. Recent genetic association studies reported the effects of 1,25(OH)_2_D on gene expression and its influence on some epigenetic modifications [8],[9],[10]. It is estimated that VDR activation may regulate directly and/or indirectly the expression of a very large number of genes (0.5–5% of the total human genome i.e., 100–1250 genes) [1],[9],[10].

Our knowledge of both the molecular events controlling regulation of gene transcription by the VDR and the target genes underlying its physiological functions has benefited tremendously in the last decade by the advent of techniques for genome-wide analysis of transcriptional regulation[11]. In vitro microarray studies in cultured human cells provided insights into some target genes underlying the broad physiological actions of 1,25(OH)_2_D_3_ [11],[12]. These in vitro findings have helped provide a molecular genetic basis for the wide variety of biological and physiological responses regulated by 1,25(OH)_2_D_3_ [9],[11],[12].

Early microarray studies performed in squamous carcinoma cells derived from a tumor of the oral cavity revealed that an analog of 1,25(OH)_2_D_3_ regulated the expression of numerous genes implicated in immune function[12]. Genome-wide microarray analysis of 1,25(OH)_2_D_3_-treated human osteoblasts revealed an interplay of critical regulatory and metabolic pathways and supported the hypothesis that 1,25(OH)_2_D_3_ can modulate the coagulation process through osteoblasts, activate osteoclastogenesis through inflammation signaling and modulate the effects of monoamines by affecting their reuptake[11].

Although there have been numerous observations of vitamin D deficiency and its links to chronic diseases, a study on how a person's vitamin D status and improvement with vitamin D supplementation affects genomic expression in vivo in humans has not been reported. The objective of this study was to determine the effect of vitamin D status and subsequent vitamin D supplementation on broad gene expression in the white blood cells collected from healthy adults before and two months after daily supplementation with either 400 or 2000 IU vitamin D_3_. **(**Trial registration: ClinicalTrials.gov NCT01696409; http://clinicaltrials.gov/ct2/show/NCT01696409?term=Holick&rank=4).

## Methods

### Trial design

This research study was a randomized, controlled, double-blind, investigator-initiated, single center pilot trial. The study protocol approved by the Boston University Medical Campus Institutional Review Board and registered in ClinicalTrials.gov with Identifier: NCT01696409. The protocol for this trial and supporting CONSORT checklist are available as supporting information; see Checklist S1 and Protocol S1. A total of 11 adult subjects were recruited for this study. However, two subjects dropped out before using supplement and the nine subjects were randomly assigned to 1 of 2 groups (Group I and Group II) using a computer-generated simple randomization scheme. Four subjects were assigned to Group I to receive 400 IU/d of vitamin D_3_ for 8 weeks, and five subjects were assigned to Group II to receive 2,000 IU/d of vitamin D_3_ for 8 weeks. After signing a consent form and receiving the supplement, one subject from group I refused further participating in the study. The eight remaining subjects included 3 subjects in group I and 5 subjects in group II who received vitamin D_3_ for 8 weeks and were assigned to the final analysis for studying the effect of vitamin D supplementation on broad gene expression. Based on vitamin D status at baseline, 4 subjects were vitamin D deficient and the other four subjects were insufficient or sufficient. Comparing gene expression in these two groups (deficient vs. insufficient or sufficient) led us to study the effect of vitamin D status on broad gene expression. The consent form was signed before the subjects were given the supplement so that we could evaluate the influence of vitamin D status and vitamin D_3_ supplementation on genome wide expression in white blood cells. None of the recruited subjects refused to give consent. To minimize sun exposure as a confounding factor, the study was conducted in the winter months. Study visits were conducted at the General Clinical Research Unit (GCRU) at Boston Medical Center. Subject demographics and total 25(OH)D levels before and after vitamin D_3_ supplementation are shown in Table 1.

**Table 1:** Subject demographics and total 25(OH)D levels before and after 400 IU/d or 2000 IU/d of vitamin D_3_ supplementation for 8 weeks.

	400 IU/d (N = 3)	2000 IU/d (N = 5)
Sex (Women)	2	1
Age (years)	27.3±2	26±5.1
25(OH)D levels before supplementation (ng/ml)	18.3±1.1	24±10.7
25(OH)D levels after supplementation (ng/ml)	24±5.2	33.8±7.8

Demographic information including sex, average age and 25(OH)D levels are included (mean ± standard deviation).

### Study Subjects

Healthy, non-patient English speaking adult males and females of all ethnic groups age 18 and older were recruited for this study. Before any study procedures were initiated for any subject in this study, a written informed consent was properly executed and documented as approved by the Boston University Medical Campus Institutional Review Board. A Federal wide Assurance Number FWA00000301 has been approved for Boston University Medical Center. The informed consent form is available as supporting information; see Informed consent form. The exclusion criteria included: pregnant/lactating women; current or recent history of hepatic or renal disease; supplementation of greater than 400 IUs vitamin D_2_ or vitamin D_3_; current antiseizure medications or glucocorticoids; tanning for more than 8 hours within the past month; history of intestinal malabsorption; and unwillingness to consent to the study.

### Visits

During the subjects' first and second visits to the GCRU, demographic data, body weight, height, body mass index (BMI), past vitamin D use, urine pregnancy test (females only), diet, current medication usage and/or anticipated medication usage during the study period were collected on data collection forms. The subjects also received a bottle containing vitamin D_3_ capsules that contained either 400 IUs or 2000 IUs of vitamin D_3_ for the 8-week period. The vitamin D_3_ supplements were produced by Tishcon Laboratories (Westbury, NY 11590) and were found to have contained stated vitamin D content as previously described [13].

The second visit occurred 8 weeks after the first visit. Subjects returned with their vitamin D_3_ bottles and the investigators counted how many capsules remained and determined compliance.

### Blood sample collection

Ten ml of blood was also collected at each visit and sera were collected for evaluation at Quest Diagnostics and the BUMC Corelab and utilized to determine serum levels of 25(OH)D by liquid chromatography tandem mass spectroscopy as previously described [14]. Another 10 ml of blood was collected to obtain a buffy coat including white blood cells and platelets.

The white blood cells were collected to purify total RNA, including small RNAs. Purified RNA was stored at -86 degrees Celsius and sent for analysis to the Boston University Pulmonary Center's Microarray Resource Facility.

### Microarray Data Acquisition and Preprocessing

All procedures were performed at the Boston University Microarray Resource Facility as described in the GeneChip® Whole Transcript (WT) Sense Target Labeling Assay Manual (Affymetrix, Santa Clara, CA). Total RNA was isolated using QIAGEN's RNeasy kit as described in manual (Qiagen, Valencia, CA). For each sample, integrity was verified using RNA 6000 Nano Assay RNA chips run in Agilent 2100 Bioanalyzer (Agilent Technologies, Palo Alto, CA). RNA was reverse transcribed using Whole Transcript cDNA Synthesis kit (Affymetrix, Santa Clara, CA). The obtained antisense cRNA was purified using GeneChip Sample Cleanup Module (Affymetrix, Santa Clara, CA), and used as a template for reverse transcription (Whole Transcript cDNA Synthesis kit, Affymetrix, Santa Clara, CA) to produce single-stranded DNA in the sense orientation. During this step dUTP was incorporated. DNA was then fragmented using uracil DNA glycosylase (UDG) and apurinic/apyrimidinic endonuclease 1 (APE 1) and labeled with DNA Labeling Reagent that is covalently linked to biotin using terminal deoxynucleotidyl transferase (TdT, Whole Transcript Terminal Labeling kit, Affymetrix, Santa Clara, CA). IVT and cDNA fragmentation quality controls were carried out by running an mRNA Nano assay in the Agilent 2100 Bioanalyzer.

The labeled fragmented DNA was hybridized to the Gene Array 1.0ST for 16–18 hours in GeneChip Hybridization oven 640 at 45°C with 60 rpm rotation. The hybridized samples were washed and stained using Affymetrix fluidics station 450. The first stain used was streptavidin-R-phycoerythrin (SAPE) followed by signal amplification using a biotinilated goat anti-streptavidin antibody and another SAPE staining (Hybridization, Washing and Sataining Kit, Affymetrix, Santa Clara, CA). Microarrays were immediately scanned using Affymetrix GeneArray Scanner 3000 7G Plus (Affymetrix, Santa Clara, CA). The resulting CEL files were summarized using Affymetrix Expression Console (current version 1.1). Robust Multi-Array Average (RMA) algorithm was used to generate gene-level data.

### Real time –PCR verification

The cDNA was then used to check for the gene expression of four specific genes, via qRT-PCR. This was done in duplicate, so that a total of 32 samples were tested for each gene (2×16 samples). This was accomplished by adding 2.5 uL of each of the 16 samples into a 96-well plate. 22.5 uL of the TaqMan Master Mix was then added to each sample in the plate. The TaqMan Master Mix (per sample) included 12.5 uL of 2× TaqMan, 1.25 uL of oligonucleotide ribosomal RNA (rRNA), and 8.75 uL H2O. The expression of an 18S rRNA was also tested. Since the 18S gene is expressed in all cells it was used as an endogenous control. Each 96-well plate was covered and centrifuged at 1,500 rpm and 4oC for 5 minutes. After spinning the plate was put into the Applied Biosystems StepOnePlus Real-Time PCR system to check for gene expression. The total run time for each plate in the StepOnePlus was about 40 min. The raw data was saved on a computer and later analyzed.

### Searching for candidate vitamin D response elements

To investigate the role of VDR binding in vitamin D mediated gene expression, we searched for VDR binding domain within 60 kb (especially in 5 kb) of the transcriptional start site (TSS) of vitamin D responsive genes. From the 291 genes influenced by vitamin D_3_ supplementation 17 genes that were most affected by vitamin D_3_ supplementation were selected for vitamin D response element (VDRE) analysis.

For our analysis we first evaluated known VDREs that are shown in Table S1. These motifs were entered in to the CLC workbench (version6.2) as new motifs to search for novel VDREs as shown in Table S2.

This structural study was also performed on 12 housekeeping genes. The housekeeping genes were used as negative controls for candidate VDREs. Expressions of these housekeeping genes after vitamin D_3_ supplementation were not changed. There were no sequences of candidate VDREs in 100 kb upstream of the TSS of these housekeeping genes. The list of these housekeeping genes and the region of the study is summarized in Table S3.

As positive controls the known target genes for 1,25(OH)_2_D_3_ and reported VDREs in them were used (Table S1). In some VDRE candidates the same sequence was found like the VDRE in the RANKL gene (Table 2). In some genes the first hexameric motif was similar to the first part in VDRE in the one target gene and the second part similar to the second part of the another genes' VDRE. The similarity of the candidate VDREs with the known VDREs are demonstrated in Table 2. For example the candidate VDRE in coatomer protein complex, subunit beta 2 (COPB2), a gene that was stimulated at least 1.5 fold by vitamin D_3_ supplementation, had two hexameric binding motifs associated with the VDRE. The first binding motif (TGAACT) was similar to the VDRE in receptor activator of NFkB ligand (RANKL) and the second binding motif (AGGTGA) was similar to VDRE in cytochrome P450, family 24, subfamily A, polypeptide 1 (25-hydroxyvitamin D-24-hydroxylase, CYP24A1).

**Table 2:** The motif sequences of 17 genes that were most response to vitamin D_3_ supplementation that is identical or similar to other known VDRE sequences.

Gene	Effect of vitamin D_3_ supplementation		Sequence	Position of VDRE	Similarity to known VDRE
	Effect on expression	Fold changed			
TIA1	Increased	26	GGTTCAagcAGTTCT	−19259	RANKL
ZNF287	Increased	6.8	GGGCGAgcaAGGGGA	−33162	Similar to MIS
Y-RNA	Increased	2	GGGTTAtggAGACCA	−13579	Similar to Insulin receptor
			AGACCAgcaAGGGCA	−15357	C-fos
			AGGGGAgtgGGCTCA	−5398	Lrp5
			AGAACTgttTGAACT	2000	RANKL, CYP24A1
			AGGGCAatgGGCTCA	−17578	C-fos,Lrp5
			TGAACTgccGGGCCA	−16195	RANKL, CYP24A1
			GGGGGAggcGGGCGA	−16589	Similar to Osteocalcin
CETN3	Increased	1.9	GGGTTCcacAGTTCT	−7715	RANKL,Lrp5
			AGGCGAggcAGGGGA	257	Similar to MIS
			AGACCAgctGGGGCA	7743	Osteocalcin, Insulin Receptor
MINPP1	Increased	1.6	AGGGCAgggGGCGGG	−33	C-fos
			AGGTTAgttGGGTCA	−7722	Insulin receptor
			GGTTCAagcAGTTCT	−15303	RANKL, Osteopontin, LRP5
PUS3	Increased	1.6	AGGGCAagaGGGGCA	−1027	Osteocalcin,Ros
			GGTTCAagcAGTTCT	−11623	RANKL, osteopontin
			AGGGCAaaaAGTTCG	−23238	C-fos
			AGGCCAacaGGGGCA	−28062	Osteocalcin, Insulin receptor
			AGGACTacaGGGACA	−38442	hWise,MIS
ZDHHC16	Increased	1.5	GGTTCAagcAGTTCT	−43597	RANKL
PTRH2	Increased	1.5	TCATTCataAGGGCA	−6751	RANKL, C-fos
NUDCD1	Increased	1.5	GGTTGAagaGGGTGA	−18094	Similar to RANKL
COPB2	Increased	1.5	TGAACTcttAGGTGA	−28579	RANKL, CYP24A1
TRIM27	Increased	1.5	GGTTCAagcAGTTCT	−10940	RANKL, osteopontin, Lrp5
HSPH1	Increased	1.5	GGGGTAatcCAGACA	−185	PTH
			AGGCCAgttGGGGCA	−13744	Osteocalcin, Insulin receptor
			GGGTCAgacAGGGCA	−15275	Insulin receptor,Ros
KEAP1	Increased	1.5	AGGCCAaggGGGGCA	−12008	Osteocalcin, Insulin receptor
			AGGGCAatgGGCTCA	−17578	C-fos,Lrp5
			TGAACTgccGGGCCA	−16195	RANKL, CYP24A1
			GGGGGAggcGGGCGA	−16589	Similar to Osteocalcin
CD83	Decreased	2	AGTTCAaacAGTTCT	2237	RANKL, CYP24A1
			GGGCCAgaaGGGTTA	−12371	Similar to Osteocalcin
			GGTTCAagcAGTTCT	−72447	RANKL, Osteopontin, LRP5
			AGGTCAgagAGGTGA	−41941	CYP24A1
			AGGTGAgaaAGTTCA	−27226	RANKL, CYP24A1
			AGTTCAgagGGGTGA	−84403	RANKL, CYP24A1
			GGGTGAtctAGTTGA	−106730	CYP24A1
			GGGTCAtgaGGGTCA	−121008	Insulin receptor
TNFAIP3	Decreased	1.5	AGGACAaatGGGACA	−71377	hWise
			TGCCCTgacTGGTCT	−115872	C-fos
			AGGTGAatgGGGTGA	−108862	CYP24A1
			AGTTGAtgaAGTTGA	−142558	CYP24A1
NFKBIA	Decreased	1.5	GGGTGAcatGGGTGA	−31881	MIS
TNNI3K	Decreased	1.6	GGGTCAgaaACAACC	−51	Insulin receptor, RANKL
			AGGTGAaacAGGTCA	−29062	CYP24A1
			AGGGCGgaaGGGGGA	−23892	CYP24A1

The positions and sequences of nucleotide motifs from 5′ upstream to the transcriptional start site for 17 genes identified as being most affected by vitamin D_3_ supplementation. The similarity of the candidate VDREs with known VDREs is shown.

### Sample size estimation

The sample size in this pilot study was estimated based on the pilot funding and on results from previous report of vitamin D impact on broad gene expression in vitro model [11],[12], it was estimated that vitamin D could reduce expression in some genes or increase expression in other genes between one to three fold. A two-sided risk of type 1 error, α, of 0.05; a type 2 error risk, β, of 20%; (power of 80%), changing relative expression from 1.5 to 3, standard deviation of 1(0.3 to 1) and equal group size, the sample size (N) for was calculated to N = 4 for each group.

The actual inclusion of 8 subjects in the two supplemented groups or based on their vitamin D status resulted in a power of 57%–92% for the effect of vitamin D status and vitamin D supplementation on expression of TRIM27, CD83, COPB2, YRNA and CETN3.

### Data Analysis

The sample size for this study was 8 subjects. Since each subject had two samples (baseline and follow-up visits), a total of 16 samples were analyzed and linked to experimental factors of supplementation and time. All 16 arrays were normalized with the RMA method as described above. For data quality control regarding the similarity of the samples within and between the groups, the Principal Component Analysis (PCA) method was used. Microarray data normalization was conducted to correct for the mean intensity for each array. To identify the differentially expressed genes before versus after supplementation and between two kinds of supplementation (400 IUs or 2000 IUs), a 2-way ANOVA in the linear model was applied. To assess the results, the p<0.01 was used as a cutoff due a small sample size. Also all subjects were classified based on basal levels of 25(OH)D. Four subjects were vitamin D deficient with 25(OH)D of 16±3.8 ng/ml and the other four subjects were insufficient or sufficient [2] with a 25(OH)D of 27.6±5.4 ng/ml.

It is standard practice in biostatistics to use a p value threshold of 0.05 for the decision as to whether a difference is significant or not. This p value is the probability of getting a false positive result, so on average we would expect to get a false positive result about once every 20 times the test was used [15]. Thus in this study the level of statistical significance was defined as alpha <0.01 i,e. there was a 1% maximum chance of incorrectly rejecting the null hypothesis that there is no association between vitamin D supplementation and genetic expression. The FDR <0.1, (False Discovery Rate) was applied to a list of genes, not any particular gene. We conducted appropriate correction for multiple testing that included using a 1.5 fold change of gene expression combined with false discovery rate (Fdr) < 0.1 in our main analysis. Using a 1.5 fold change of gene expression combined with ANOVA in our subgroup analysis. Using P <0.01 for decreasing false positive and technique validation with the real replicate time PCR. Significant enrichment of GO biological process categories were tested for using EASE software (version 2.0) with P<0.05 [16].

## Results

### Demographic and other baseline characteristics

**Figure 1:**
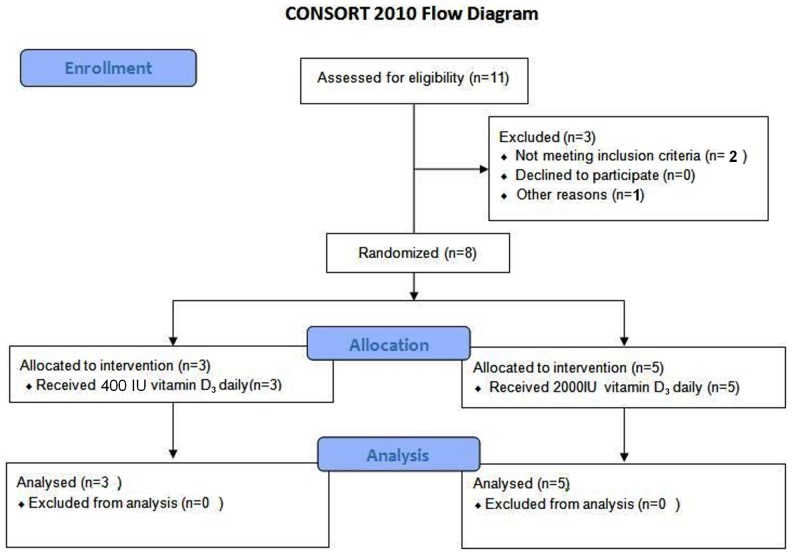
Flow Diagram of Study Subjects.

Eight subjects who met the inclusion/exclusion criteria were enrolled. No recruited subjects refused to give consent. Sixteen microarrays (baseline and after 2 months of vitamin D_3_ supplementation) from eight subjects (3 women) passed the quality control filters and normalized with the RMA method. Mean of age, BMI and serum 25(OH)D levels were 26.5±4 years, 27±5.9 kg/m2 and 21.8±8.6 ng/ml respectively and all of them were white. Three participants received 400 IUs of vitamin D_3_ daily and five participants received 2000 IUs of vitamin D_3_ daily (Figure 1). After eight weeks of vitamin D_3_ supplementation serum 25(OH)D levels in the group that received daily 2000 IUs had a 2-fold increase (9.8±4.9 ng/ml) compared to subjects who received and had an increase of 5.6±4.9 ng/ml (Table 1).

### Impact of vitamin D_3_ supplementation on expression of genes in human white blood cells

**Figure 2:**
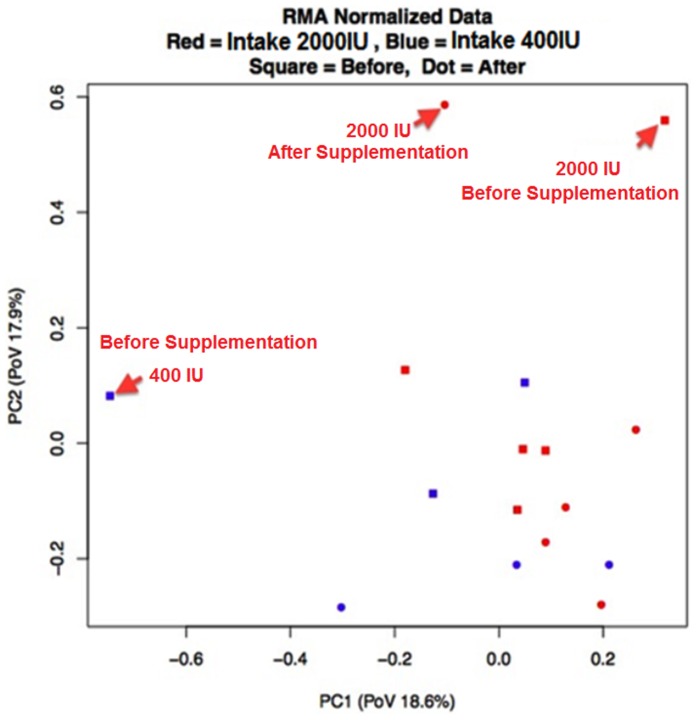
Principal Component Analysis across 16 microarray samples. There is no grouping of samples along the first or second principal components (representing 18.6% and 17.9% of the variance in gene expression, respectively) based on the expression of these genes. Sample types of each group before or after vitamin D_3_ supplementation are color-coded for the dose of vitamin D_3_ supplementation. Red =  2000 IUs and blue =  400 IUs (PoV  =  Possibility of Variance.)

**Figure 3:**
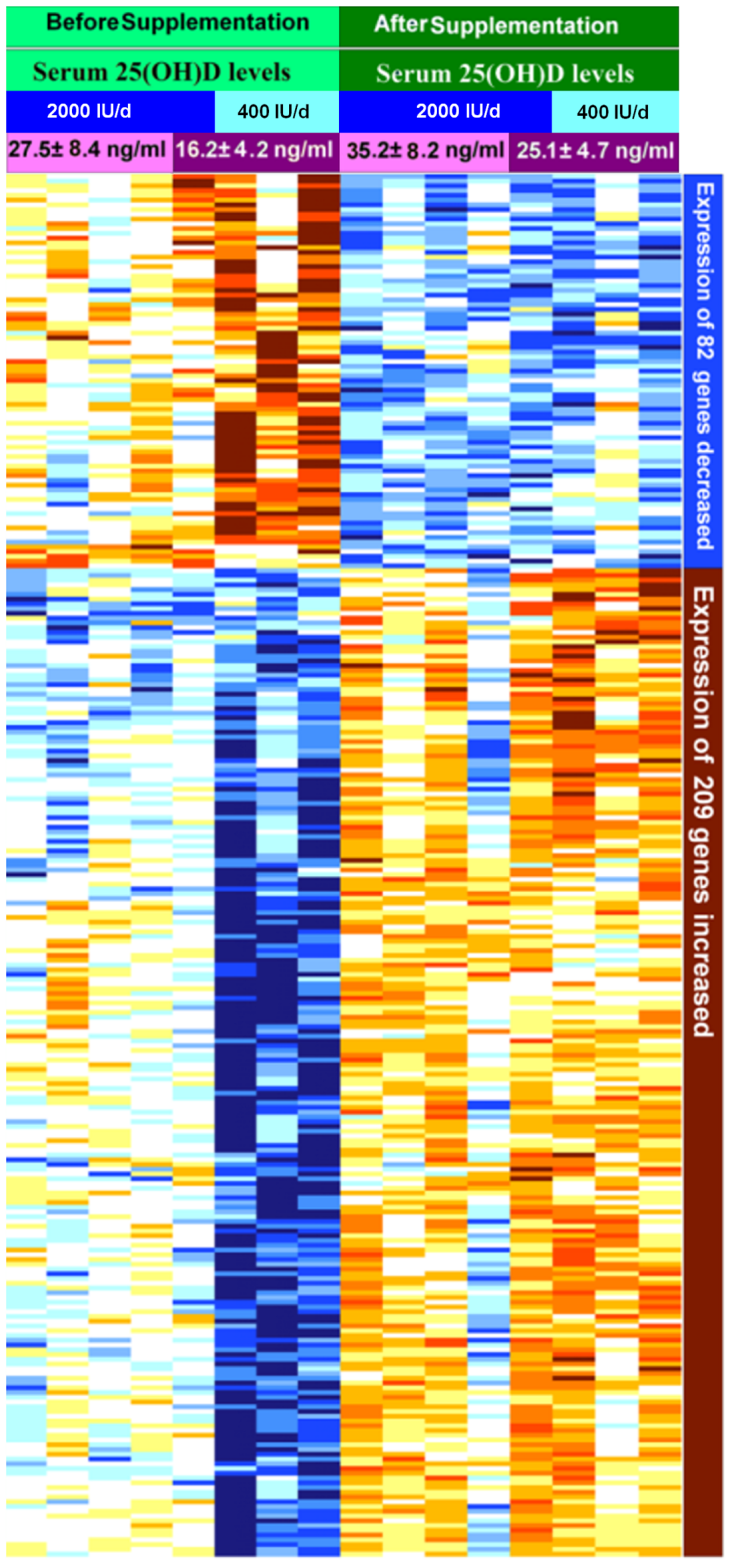
Heatmaps of vitamin D responsive genes whose expression levels change after 2 months vitamin D_3_ supplementation. Before supplementation (light green) four subjects were vitamin D deficient with 25(OH)D of 16.2±4.2 ng/ml (dark purple) and the other four subjects were insufficient or sufficient with a 25(OH)D of 27.5±8.4 ng/ml(light purple). After supplementation (dark green) serum levels of 25(OH)D in vitamin D insufficient/sufficient subjects increased to 35.2±8.2 ng/ml (light purple) and in the vitamin deficient subjects increased to 25.1± 4.7 ng/ml(dark purple). Two groups of gene-expression changes are seen based on stimulation (brown) or inhibition (blue) of gene expression post vitamin D_3_ supplementation. (Colors ranged from blue to brown; High expression  =  brown, average expression  =  white, low expression  =  blue). Clustering of the 291 genes affected by vitamin D_3_ supplementation was based on stimulation (brown) or inhibition (blue) of gene expression. The list of the 291 genes is shown in Table S1.

To explore gene-expression relationships between and within the 400 IU and 2000 IU groups principal component analysis (PCA) was performed. Total variability of individual chips after normalization is illustrated in Figure 2. There wasn't a significant difference in order to explore gene-expression relationships between and within the 400 IU and 2000 IU groups. Regarding all participants, with false discovery rate (Fdr)<0.1, and a 1.5 fold change, 291 genes were found to have a statistically significant difference in expression from baseline to follow-up after vitamin D_3_ supplementation (Figure 3). The list of these 291 genes is shown in Table S4. There was at least a 1.5 fold inhibition of 82 genes (top ∼30% of the heat map) whose expression was dramatically reduced and at least a 1.5 fold induction of 209 genes (bottom ∼70% of the heat map) whose expression was significantly increased after supplementation with either 400 or 2000 IU of vitamin D_3_ for 2 months.

**Figure 4:**
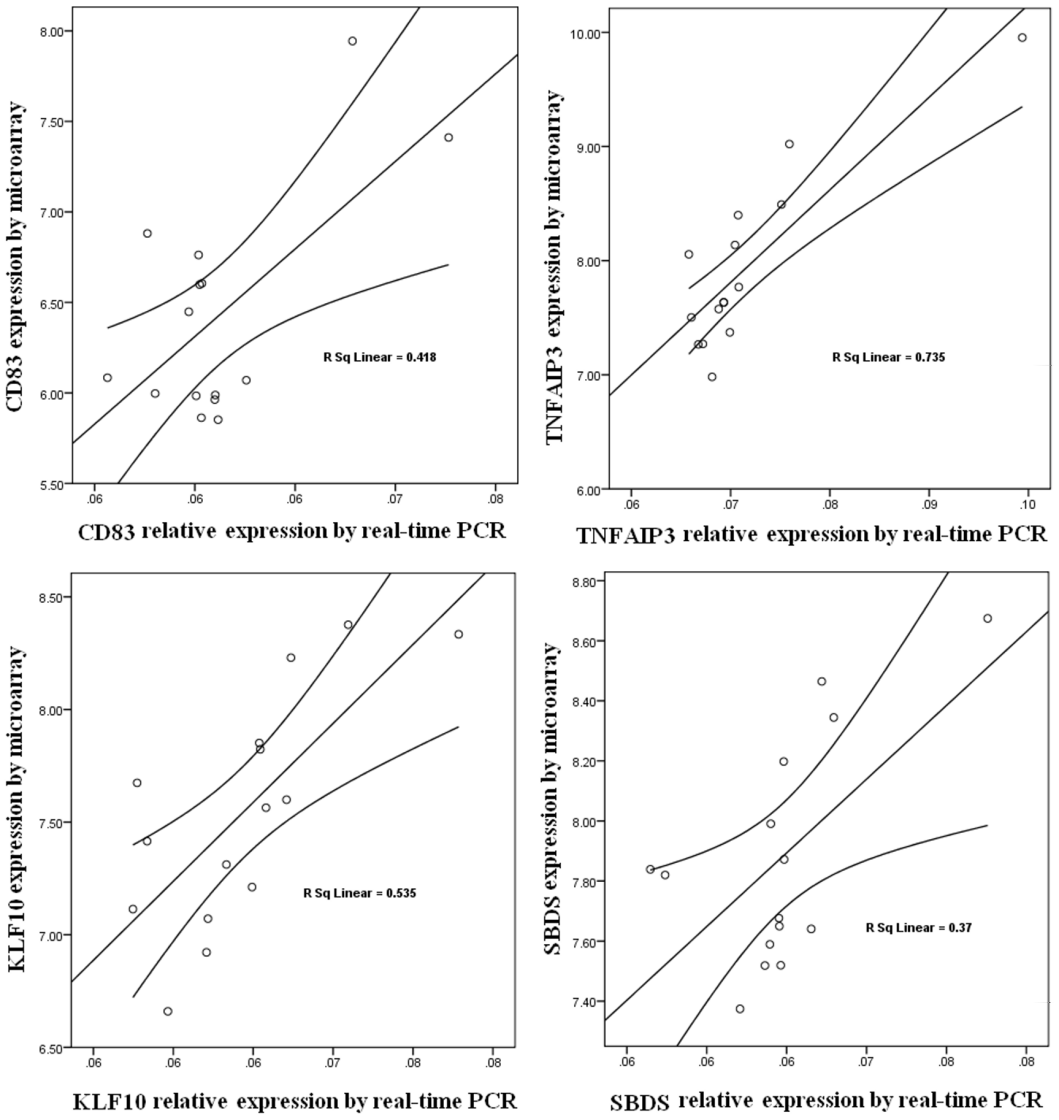
Verification of microarray gene expression by Real-time PCR. For verification of gene expression real-time PCR was performed for four genes including CD83, TNFAIP3, KLF10 and SBDS. Relationship between two sets of data from microarray and real-time PCR is shown by linear regression with 95% mean prediction interval. The results showed the relative expression of these genes was consistent with the expression observed from the broad gene expression by microarray.

For verification candidate of gene expression changes real-time PCR was performed for four genes including CD83, TNFAIP3, KLF10 and SBDS (Figure 4). The results showed gene expression changes concordant with those observed by microarray.

### The effect of vitamin D status on gene expression

**Figure 5:**
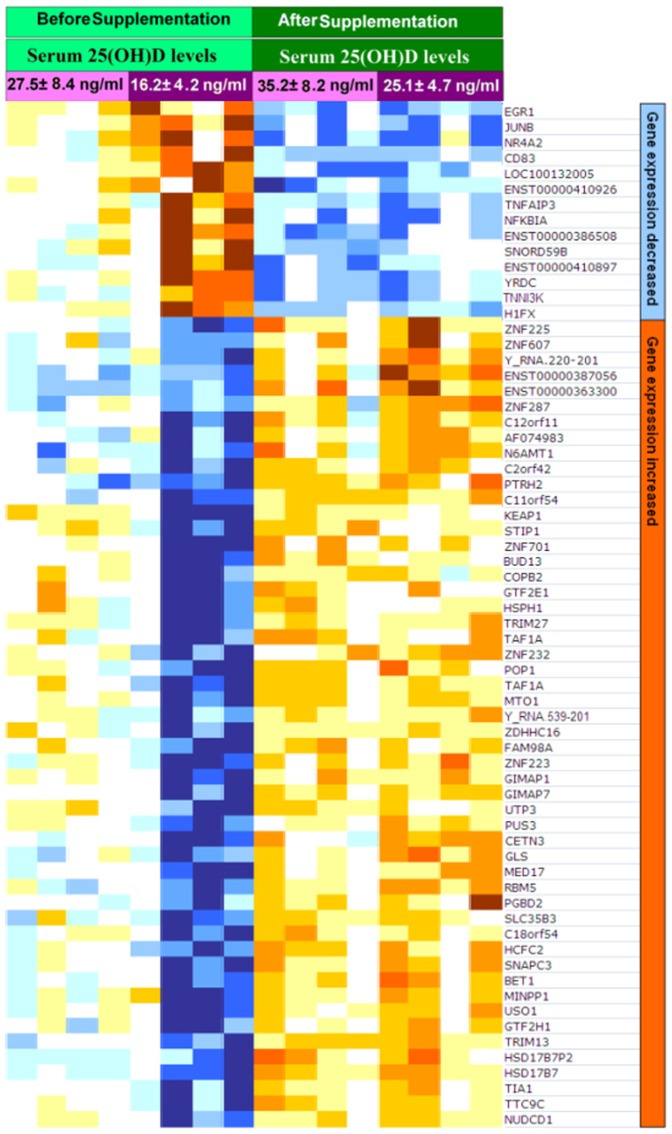
Heatmaps of vitamin D responsive genes affected by vitamin D status. Before supplementation (light green) four subjects were vitamin D deficient with 25(OH)D of 16.2±4.2 ng/ml (dark purple) and the other four subjects were insufficient or sufficient with a 25(OH)D of 27.5±8.4 ng/ml(light purple). After supplementation (dark green) serum levels of 25(OH)D in vitamin D insufficient/sufficient subjects increased to 35.2±8.2 ng/ml (light purple) and in the vitamin deficient subjects increased to 25(OH)D of 25.1±4.7 ng/ml(dark purple). Two groups of gene-expression changes are seen based on stimulation (brown) or inhibition (blue) of gene expression post vitamin D_3_ supplementation. (Colors ranged from blue to brown; High expression  =  brown, average expression  =  white, low expression  =  blue).Expression of 66 genes before supplementation was significantly different in the vitamin D deficient group (dark purple) compared to the vitamin D insufficient/sufficient group (light purple). Clustering of the 66 genes affected by vitamin D status and vitamin D_3_ supplementation was based on stimulation (brown) or inhibition (blue) of gene expression.

A subgroup analysis of participants was evaluated based on the baseline serum 25(OH)D levels (Figure 3 and 5) in order to determine what if any influence vitamin D status had on the basal expression of the 291 genes identified as being affected by 2 months of vitamin D_3_ supplementation. Four subjects were vitamin D deficient with 25(OH)D 16.2±4.2 ng/ml (range 10–19 ng/ml) and the other four subjects were insufficient or sufficient with a 25(OH)D of 27.5±8.4 ng/ml (range 22–40 ng/ml).

This subgroup analysis of the baseline gene expression for the 291 genes (Figure 3) in the vitamin D deficient group compared to the vitamin D insufficient/sufficient group revealed that, expression of 66 genes were significantly different between the two groups (p<0.01 and fold change>1.5). (Figure 5) There was at least a 1.5 fold increase in gene expression (brown-orange) of 14 genes and at least a 1.5 fold decrease in the expression (yellow-white) of 52 genes in the vitamin D deficient adults compared to those who were vitamin D insufficient or sufficient at baseline (Figure 5). After vitamin D_3_ supplementation the serum 25(OH)D increased from 16.2±4.2 to 25.1±4.7 ng/ml and 27.5±8.4 to 35.2±8.2 ng/ml in the adults who were vitamin D deficient and vitamin D insufficient or sufficient respectively. After vitamin D_3_ supplementation gene expression in the vitamin D deficient group was similar to vitamin D insufficient/sufficient group and there was no longer a significant difference between two groups in the expression of these 66 genes.

### Structural evidence for the effect of vitamin D_3_ supplementation on gene expression

To learn which of these genes affected by vitamin D_3_ supplementation contained VDR binding domains near the transcriptional start site (TSS), we performed a VDRE analysis as described in Materials and Methods.

Of the 66 genes that were influenced by at least 1.5 fold in their expression by the baseline serum 25(OH)D concentration, 17 of these genes that were significantly changed after vitamin D_3_ supplementation in both deficient and insufficient/sufficient groups (p<0.01) were selected for VDRE analysis.

The details of searching for candidate VDRE sequences is explained in Methods and shown in Table S1-3. We found at least one candidate VDRE in the upstream region within 30 kb of the TSS in these 17 genes (Table 1). For example, the candidate VDRE in coatomer protein complex, subunit beta 2 (COPB2), a gene that was stimulated at least 1.5 fold by vitamin D_3_ supplementation, had two hexameric binding motifs associated with the VDRE. The first binding motif (TGAACT) was similar to the VDRE in receptor activator of NFkB ligand (RANKL) and the second binding motif (AGGTGA) was similar to the VDRE in cytochrome P450, family 24, subfamily A, polypeptide 1 (25-hydroxyvitamin D-24-hydroxylase, CYP24A1).

**Figure 6:**
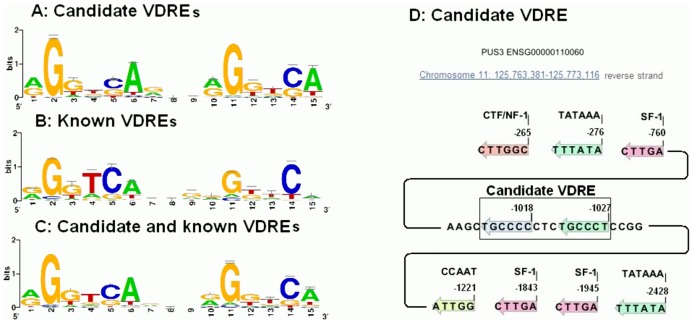
Sequence of candidate VDREs compared with known VDREs. (A) The candidate sequences of VDREs (B), motifs created based on known VDRE sequences previously reported and (C) motifs based on the sum of these sequences and (D) the location of candidate VDREs of pseudouridylate synthase 3 (PUS3) and the location of other transcription regulation sites in this gene including TATA box, SF1and CCAAT. The major structure of candidate VDREs are based on the consensus sequence RGKTSA (R  =  A or G, K  =  G or T, and S  =  C or G).

The motif sequence for VDR/RXR hetrodimeric binding sites is shown in Figure 6. The motif sequence of candidate VDREs (6A) are compared with known VDREs (6B). All of these sequences are summarized in one motif sequence (6C). The location of other transcription factor binding sites are shown in Figure S1. These are associated with known steroidogenic factor 1 (SF-1), CTF1/nuclear factor 1 (NF1), CCAAT enhancer binding protein-β (C/EBPβ), NF-KB and RNA polymerase (TATA box) motifs. For example pseudouridylate synthase 3 (PUS3), a gene that was stimulated 1.6 fold by vitamin D_3_ supplementation has five VDREs (Table 2) that one of them is shown in Figure 6D located at position -1027, the TATA box located at -276 and location of other transcription factor sites near this VDRE was determined (6D).

This study was also performed on 12 housekeeping genes to serve as negative controls. There were no sequences of candidate VDREs in 100 kb upstream of TSS of these housekeeping genes (Table S3). The expression of these housekeeping genes after vitamin D_3_ supplementation was not changed.

### Biological functions for vitamin D responsive genes

**Figure 7:**
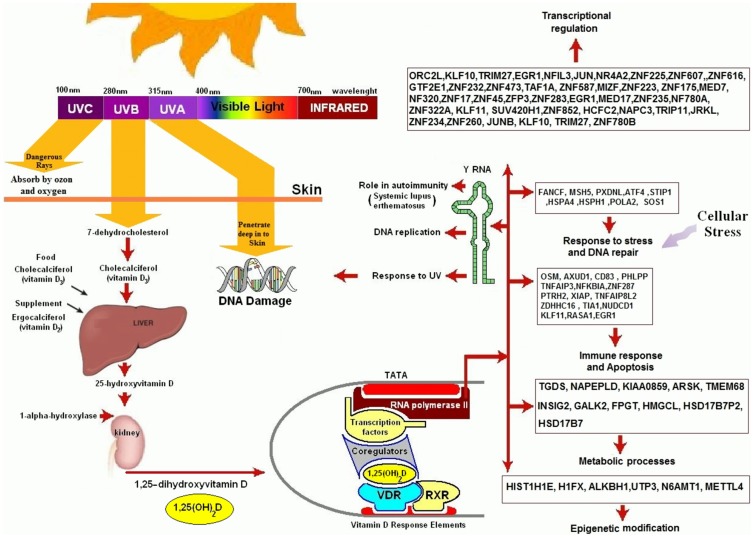
Biological functions for genes whose expression levels were altered after 2 months of vitamin D_3_ supplementation. After receiving vitamin D_3_ supplementation we identified 291 genes whose expression was significantly decreased or increased. Some of these genes influence several pathways that are involved in response to stress and DNA repair, DNA replication, immune regulation, epigenetic modification, transcriptional regulation and other biological functions. In addition vitamin D_3_ supplementation influenced the expression of Y RNA and CETN3 that are involved in DNA repair in response to UVR exposure.

An analysis of the 291 genes affected by the vitamin D_3_ supplementation was associated with at least a 1.5 fold induced expression of genes related to 81 pathways and at least a 1.5 fold inhibition of genes affecting 88 pathways (Table S5, S6)(p<0.05). The most relevant biological functions resulting from these changes in gene expression by vitamin D_3_ supplementation are listed in Table 3 and the complete list is in Tables S5 and S6. Gene ontology analysis showed that the differentially expressed genes were significantly enriched with those associated with immune functions, transcriptional regulation, cell cycle activity, DNA replication and response to stress (Figure 7).

**Table 3:** List of biological functions of the 291 genes whose expression was influenced by vitamin D_3_ supplementation.

Biological functions	Gene symbol
Apoptosis and immune response	OSM, AXUD1, CD83, PHLPP, TNFAIP3,NFKBIA,ZNF287, PTRH2, XIAP, TNFAIP8L2, ZDHHC16, TIA1,NUDCD1, KLF11,RASA1,EGR1
Mineralization and bone development	JUND, SBDS, ZNRD1,MINPP1
Transcriptional regulation	ORC2L,KLF10,TRIM27,EGR1,NFIL3,JUN,NR4A2,ZNF225,ZNF607,ZNF780B,ZNF616RASA1,ZNF397,ZNF284,ZFP62,HOMEZ,ZNF701,GTF2E1,ZNF232,ZNF473,TAF1A,ZNF587,MIZF,ZNF223, ZNF175,MED7, ZNF320,ZNF17,ZNF45,ZFP3,ZNF283, EGR1,MED17,ZNF235,NF780A,ZNF322A, KLF11, SUV420H1,ZNF852, HCFC2,NAPC3,TRIP11,JRKL,ZNF234,ZNF260,JUNB, KLF10, TRIM27
Metabolic processes	TGDS, NAPEPLD, KIAA0859, ARSK, TMEM68INSIG2, GALK2, FPGT, HMGCL, HSD17B7P2, HSD17B7
Response to stress and DNA repair	FANCF, MSH5, PXDNL,ATF4,STIP1,HSPA4,HSPH1,POLA2, SOS1
RNA processing	CCDC76, MTO1, C1orf25, PUS3, RBM5, CSTF3, ZFP36, RNASEL, NUP107, ZCCHC8,POP1,INTS7, GEMIN6
Ion and protein transporter	SLC39A7, SLC30A6, SLC30A5, COPB2, NUP43, GOPC, SLC35B3, BET1, USO1, PIGM, TRAPPC6B
Biological process	TGDS, NAPEPLD, KIAA0859, ARSK, TMEM68, INSIG2, GALK2, FPGT, HMGCL, HSD17B7P2, HSD17B7
Epigenetic modification	HIST1H1E, H1FX, ALKBH1,UTP3, N6AMT1, METTL4
Cell cycle and DNA replication	FGF5, IRS2, MIS12, C10orf2, ORC2L, HELB, CUZD1, KIAA1009, POLA2, CETN3,CEP110, POLA2, PTP4A1
Signal transduction	GRASP, GNRH1, TAS2R4, TAS2R3, CXCR7, RIC8B, SOS1, BBS10
Protein modification	PIM3, PTP4A1, RNF139, PDP2, GGCX, PPID, TTC9C, SIK1, STK38
Development and cell differentiation	PDE4DIP, TUBD1, KEAP1, BBS7, UTP3, C11orf73, MKKS, BPNT1,NOC3L

## Discussion

This genome-wide expression analysis provides the first insight into the global transcriptional activity that underlies the effects of vitamin D status and vitamin D_3_ supplementation in cells on the human buffy coat that include immune cells. As shown in Figure 3, vitamin D supplementation caused at least a 1.5 fold change in the expression of 291 genes that are involved in apoptosis, immune function, transcriptional regulation, epigenetic modification, response to stress, cell cycle activity and differentiation (Table 3). This finding is consistent with previous in vitro studies that showed 1,25(OH)_2_D_3_ directly or indirectly controlled more than 200 genes, including genes responsible for the regulation of cellular proliferation, differentiation, angiogenesis and immunomodulatory activities on both innate and adaptive immune responses [3],[4],[6]. Our observations support previous reports that have estimated that VDR activation may regulate directly and/or indirectly the expression of a very large number of genes (0.5–5% of the total human genome i.e., 100–1250 genes) [3],[4],[8],[11],[17].

In a recent genome-wide microarray analysis of 1,25(OH)_2_D_3_-treated human osteoblasts revealed modulation of 158 genes involved in vitamin D metabolism (CYP24A1), immune function (CD14), neurotransmitter transporters (SLC1A1, SLC22A3), and coagulation [17]. A study of the human genome screened for VDREs reported 157 genes to be regulated in SCC- 25 cells, of which 126 were induced and 31 were repressed [17]. The researchers found 2,776 binding sites for the vitamin D receptor along the length of the genome. Among them included 229 genes associated with susceptibility to autoimmune disorders, and cancers including chronic lymphocytic leukemia and colorectal cancer [17]. All of these studies reporting the effect of 1,25(OH)_2_D_3_ on gene expression were in vitro studies in different cell types.

There have been two genome wide association studies that have related genomic variation on vitamin D status [18],[19]. However little is known about what effect vitamin D status has on gene expression or what happens to gene expression in response to vitamin D supplementation. We observed that 291 genes were at least a 1.5 fold stimulated or inhibited in response to vitamin D_3_ supplementation. We identified 66 genes, that were most significantly affected by the subjects' vitamin D status i e those who were vitamin D deficient with 25(OH)D of 16.2±4.2 ng/ml compared to adults who had a 25(OH)D of 27.5±8.4 ng/ml at baseline. Nineteen of these 66 genes are the same reported by in vitro studies [12], [17]. Thus we have identified an additional 47 genes that were influenced by vitamin D_3_ status.

Of these 66 genes, 17 genes whose expression significantly changed after vitamin D_3_ supplementation in both deficient and insufficient/sufficient groups were found to have novel VDREs (Table 2).

After receiving 400 IUs or 2000 IUs for 8 weeks of vitamin D_3_ dramatic changes occurred in the expression of these 66 genes while no significant change was seen in 12 housekeeping genes. We did not see a significant dose dependent difference in the alteration in gene expression 8 weeks after the adults received daily either 400 IUs or 2000 IUs vitamin D_3_. This could be due to the small number of subjects studied or that any improvement in serum 25(OH)D levels can lead to significant changes in gene expression whether the person is vitamin D deficient, insufficient or sufficient. We observed the same trend in gene expression in the subjects who received 400 or 2000 IUs vitamin D_3_ whether the baseline 25(OH)D was 16.2±4.2 or 27.5±8.4 ng/ml. There was however a trend for a larger change in the expression of these genes for the group who received 2000 IUs vitamin D_3_/d compared to the group who received 400 IUs vitamin D_3_/d. Even the subject who had a 25(OH)D of 40 ng/ml after 2000 IUs vitamin D_3_ daily for two months had at least a 1-fold change in 10 genes and at least a 0.5 fold change in the expression of 33 genes from this pool of 66 genes.

Of the 66 genes, 52 increased their expression in response to vitamin D_3_ supplementation. The greatest increases were in genes that coded for apoptosis, T Cell intracellular antigen-1 (TIA1) (26-fold), immune function, zinc finger protein 287(ZNF287) (6.8-fold), response to cellular stress, Y-RNA(2-fold), centrin3(CETN3) (1.5-fold) and heat shock 105 kDa/110 kDa protein 1(HSPH1) (1.5-fold), tRNA processing, mitochondrial translation optimization 1 homolog (MTO1)(5-fold) and pseudouridylate synthase 3 (PUS3)(2-fold), transcriptional regulation such as ZNF 701 (2.3-fold), and genes involved in DNA repair, general transcription factor IIH, polypeptide 1 (GTF2H1) (7-fold) and chromatin modification small subunit processome component, homolog (UTP3) (4-fold).

The other 14 genes decreased their expression in response to vitamin D_3_ supplementation. The greatest decreases were in genes that coded for histone modification; H1 histone family, member X (H1FX) (12-fold), transcriptional regulation; early growth response 1 (EGR1)(2.8-fold). Two of the genes that had reduced expression; the cluster of differentiation 83(CD83) (2-fold) and tumor necrosis factor alpha-induced protein 3 (TNFAIP3)(1.5-fold) that are known to affect immune function also were found to have reduced expression by real-time PCR.

Our observation that vitamin D_3_ supplementation increased serum 25(OH)D levels resulting in the suppression of CD83 (2-fold) is consistent with the observation that 1,25(OH)_2_D_3_ inhibited CD83 expression in cultured dendritic cells[20]. This suggests that local synthesis of 1,25(OH)_2_D_3_ in immune cells including macrophages[21] regulates genes that affect immune function and improve immune health resulting in reducing risk for developing autoimmune diseases such as multiple sclerosis and type 1 diabetes[20].

The role of TNFAIP3 in antiapoptotic function [22] and the association of its' mutations with Crohn's disease, rheumatoid arthritis, systemic lupus erythematous, psoriasis, type 1 diabetes [23] could explain the association of vitamin D sufficiency in the prevention of chronic inflammation and autoimmune diseases. Also vitamin D's influence on the expression of nuclear factor of kappa light polypeptide gene enhancer in B-cells inhibitor, alpha (NFKBIA)(1.5-fold) may affect immune and proinflammatory responses [24],[25].

Vitamin D_3_ supplementation resulted in a 1.5 fold increase in the expression of tripartite motif containing protein 27 (TRIM27) a gene that negatively regulates CD4 T cells by ubiquitinating and inhibiting the class II phosphatidylinositol 3 kinase C2β (PI3KC2β)2β [26]. TRIM27 over expression conferred resistance to oxidative stress, by decreasing the expression of thioredoxin binding protein-2 [26]. Also TRIM27 was as recently identified an important negative regulator of mast cells in vivo, and suggests that PI3KC2β is a potential new pharmacologic target to treat IgE mediated disease [25]–[26]. Our finding has identified a potential new pathway for vitamin D affecting the immune system, allergy risk and oxidative stress via TRIM27. Coatomer protein complex, subunit beta 2 (COPB2) was another vitamin D responsive gene in our study whose expression significantly increased (1.5-fold) after vitamin D_3_ supplementation. COPB2's role in apoptosis and tumor growth suppression [27], may help explain the association of improving vitamin D status in cancer prevention [7],[17].

Higher serum concentrations of 25(OH)D at baseline and improvement in serum concentrations of 25(OH)D with either 400 IUs or 2000 IUs of vitamin D_3_ resulted in a 2.5 fold decrease in the expression of EGR-1, a gene that is a transcriptional regulator of not only differentiation and mitogenesis but also plays an important function in vascular health [28],[29]. An analogue of 1,25(OH)_2_D_3_, calcipotriol which a potent inhibitor of keratinocyte proliferation and used for treating the hyperproliferative skin disorder psoriasis was found to inhibit EGR-1 expression in cultured human keratinocyte[30]. It has been estimated that as a transcription factor EGR-1 affects the expression of more than 300 genes [31]. Thus by altering the expression of EGR1 with vitamin D supplementation has the potential cascading effect of altering an additional 300 genes. This could help explain the observation that vitamin D can directly or indirectly influence up to 5% of the human genome [4],[17].

These data suggest that there is a continuum in gene expression in response to increasing serum 25(OH)D levels. The definitions of vitamin D deficiency and insufficiency and sufficiency are somewhat arbitrary. As shown in Figure 3, our data suggest that any improvement in vitamin D status will improve expression of genes that have a wide variety of biologic functions that are associated with cellular proliferation, differentiation, immune function, DNA repair etc whether the 25(OH)D concentration is as low as 10 ng/ml or as high as 40 ng/ml. These genes are linked to cancer, autoimmune disorders and cardiovascular disease and have been associated with vitamin D deficiency [1],[5],[6].

These results suggest that to maximize vitamin D's effect on gene expression may require even higher doses than 2000 IUs of vitamin D_3_ daily. The current observation showed the specific pattern for broad gene expression of vitamin D deficiency and significant changes with increases in serum levels of 25(OH)D. Although larger studies are required to explain the clinically relevant gene expression patterns, the present genome-wide microarray study in human buffy coat for the first time identified a wide range of critical regulatory and metabolic pathways influenced by vitamin D_3_ supplementation that supports the vitamin D immunomodulatory effects and potential role in response to stress and DNA repair.

The major limitation of the study is the small number of subjects studied and thus the results are reported as an exploratory study. Although gene expression was determined with a suitable false discovery rate only a few of the 291 vitamin D responsive genes were verified by real time PCR. Furthermore although our study did not identify actual VDR binding sites with a biologic function it does support vitamin D-responsive genes from in vitro studies [11],[12],[17] and suggests 17 potential novel candidate VDREs in vitamin D-regulated genes. This will need to confirm with experimental studies.

There are several strengths of the study that include accurately measuring serum 25(OH)D concentrations by the gold standard liquid chromatography tandem mass spectroscopy assay, comparing gene expression in the same individual at baseline and 2 months after vitamin D supplementation and performing this study in the winter when sunlight does not influence vitamin D status. An additional strength was provided by the real-time PCR analysis of two genes CD83 and TNFAIP3 from the 66 gene pool that were affected by vitamin D status and two genes KLF10 and SBDS from the 291 gene pool that were affected by vitamin D_3_ supplementation (Figure 4) that corroborated the microarray expression of these four genes (Figure 5).

In summary, this is the first report to reveal how vitamin D status and vitamin D_3_ supplementation affects gene expression in healthy adults. Nineteen of these vitamin D-induced genes have been previously reported to be regulated by 1,25(OH)_2_D_3_ in vitro and function on the immune system, apoptosis, transcription regulation and response to stress.

Vitamin D supplementation has proven skeletal health benefits [3],[4], particularly in individuals at risk for vitamin D deficiency. This study reveals for the first time molecular finger prints that help to explain some of the nonskeletal health benefits of vitamin D [2],[6].

## Supporting Information

Figure S1 **The six gene upstream regions containing candidate VDRE sequences, and other transcription factors sites for steroidogenic factor 1 (SF-1), CTF1/nuclear factor 1 (NF1), CCAAT enhancer binding protein-β (C/EBPβ), NF-KB and RNA polymerase (TATA box).** Arrows indicate direction of forward or revere strand. The VDREs are located at upstream region and disfigured by mines numbers relative to the ATG translation start site. The locations of other transcription factors binding sites are also shown.
(TIF)

Table S1 **The reported vitamin D response elements in known vitamin D target genes.** The positions and sequences of nucleotide motifs from 5′ upstream to the transcriptional start site were identified.
(DOCX)

Table S2 **Definition of new vitamin D response element motifs.** The reported vitamin D response elements and related element collected and these definitions entered in CLC workbench (version6.2) as new motifs to search VDRE.
(DOCX)

Table S3 **The housekeeping genes that used as negative control for VDREs searching.** Expression of these housekeeping genes after vitamin D supplementation was not changed. There were no sequences of candidate VDREs in 100 kb upstream of TSS of these housekeeping genes. The list of these housekeeping genes and the region of the study is summarized in the table.
(DOCX)

Table S4 **The complete list of 291 genes that affected by vitamin D3 supplementation.**
(DOCX)

Table S5 **The pathways enriched with down regulated genes after treatment (p<0.05).**
(DOCX)

Table S6 **Pathways enriched with up regulated genes after treatment (p<0.05).**
(DOCX)

Checklist S1 **CONSORT Checklist.**
(DOCX)

Protocol S1 **Trial Protocol.**
(PDF)
