# Supplementary figures and images for: Influence of Vitamin D Status and Vitamin D3 Supplementation on Genome Wide Expression of White Blood Cells: A Randomized Double-Blind Clinical Trial
Source: PLoS One. 2013 Mar 20;8(3):e58725. doi: 10.1371/journal.pone.0058725 (PMC3604145; doi:10.1371/journal.pone.0058725)

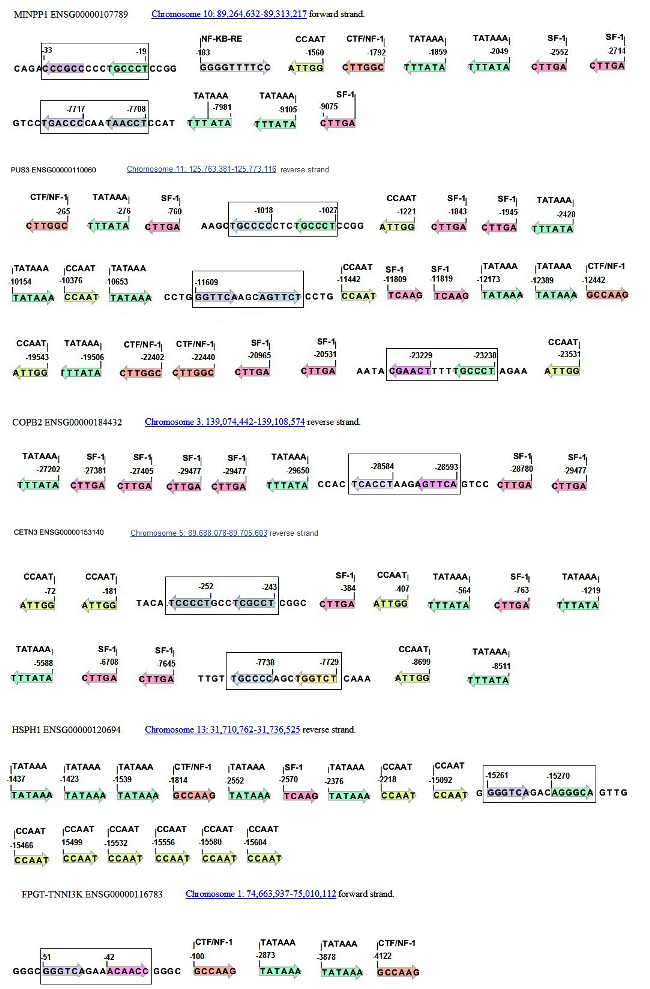

Supplement: Figure S1 — The six gene upstream regions containing candidate VDRE sequences, and other transcription factors sites for steroidogenic factor 1 (SF-1), CTF1/nuclear factor 1 (NF1), CCAAT enhancer binding protein-β (C/EBPβ), NF-KB and RNA polymerase (TATA box). Arrows indicate direction of forward or revere strand. The VDREs are located at upstream region and disfigured by mines numbers relative to the ATG translation start site. The locations of other transcription factors binding sites are also shown. (TIF) [file pone.0058725.s001.tif]
