# Supplementary material for: Influence of Vitamin D Status and Vitamin D3 Supplementation on Genome Wide Expression of White Blood Cells: A Randomized Double-Blind Clinical Trial
Source: PLoS One. 2013 Mar 20;8(3):e58725. doi: 10.1371/journal.pone.0058725 (PMC3604145; doi:10.1371/journal.pone.0058725)
Supplement: Table S1 — The reported vitamin D response elements in known vitamin D target genes. The positions and sequences of nucleotide motifs from 5′ upstream to the transcriptional start site were identified. (DOCX) [file pone.0058725.s002.docx]

| **Gene** | **Sequence** | **Position** |
| --- | --- | --- |
| Lrp5-prox | GGCTCCatgGGGTTC |  |
| Lrp5 | GGGTCActcTCATTC |  |
| CaBP 9K | GGGTGTcggAAGCCC | -488 |
| KSR-1 VDRE | GGTGCAcggAGGTCA |  |
| osteopontin | GGGTCGtatGGTTCA | -1892 |
| carbonic anhydrase II | AGGGCAtggAGTTCG |  |
| RANKL1 | TGAACTcagACAACC | _100kb |
| RANK1-2 | ACAACTtggTGACTT | -100kb |
| rankl2 | GGGTCAgcaAGGGCT | -25kb |
| rankl3 | GGTTCAtgaAGTTCT | -20kb |
| Insulin Receptor | GGGTCA(.)(.)GGGGGCA | -486 |
| MIS | GGGTGAgcaGGGACA | -395 |
| osteocalcin | GGGTGAacgGGGGCA | -499 |
| CYP24A1prox | AGGTGAgcgAGGGCG | -169 |
| CYP24A1-distal | AGTTCAccgGGTGTG | -291 |
| IGFBP-3 | GGTTCAccgGGTGCA | -3296 |
| PTH | GGTTCAaagCAGACA | -113 |
| KSR-1/2 | AATGGAatgCAGACA |  |
| hPTH | TCAACTATAGGTTCA | -121 |
| RelB | CGGTCAGGCTGGTCT | -799 |
| RelB2 | GGTTCAAGTCCCACT | -443 |
| hTRPV6^6^ | AGGTCA ttt AGTTCA | -1270 |
| hTRPV6^6^ | GGGTCA gtg GGTTCG | -2100 |
| hTRPV6^6^ | AGGTCT tgg GGTTCA | -2155 |
| hNpt2c^9^ | AGGTCA gag GGTTCA | -556 |
| hCYP3A4^20,58^ | TGAACT caaagg AGGTCA | -169 |
| hCYP3A^41^ | GGGTCA gca AGTTCA | -7.7 kb |
| hp2159 | AGGGAG att GGTTCA | -765 |
| hFOXO1^60^ | GGGTCA cca AGGTGA | -2856 |
| hWise* | AGGACA gca GGGACA | -6214 |
