# Supplementary material for: Influence of Vitamin D Status and Vitamin D3 Supplementation on Genome Wide Expression of White Blood Cells: A Randomized Double-Blind Clinical Trial
Source: PLoS One. 2013 Mar 20;8(3):e58725. doi: 10.1371/journal.pone.0058725 (PMC3604145; doi:10.1371/journal.pone.0058725)
Supplement: Table S2 — Definition of new vitamin D response element motifs. The reported vitamin D response elements and related element collected and these definitions entered in CLC workbench (version6.2) as new motifs to search VDRE. (DOCX) [file pone.0058725.s003.docx]

| GGTTCA | GGTTCA |  | Simple |
| --- | --- | --- | --- |
| AGGTCA | AGGTCA |  | Simple |
| AGGACA | AGGACA |  | Simple |
| GGGTGA | GGGTGA |  | Simple |
| ap-1 | agagtcag |  | Simple |
| CCAAT | CCAAT |  | Simple |
| ATG | ATG |  | Simple |
| nVDRE, E box | CATCTG |  | Simple |
| NF-KB-RE I | ggggttttcc |  | Simple |
| NF-KB-RE II | ggggaattcc |  | Simple |
| Hexametric | (A\|G)G(G\|T)TCA |  | Java |
| E box like | CA(.)(.)TG |  | Java |
| sox9 | ctctttgagaa |  | Java |
| SF-1 | TCAAG |  | Simple |
| SF-1 proximal | cccaaggtcgcg |  | Simple |
| TATAAA | TATAAA |  | Simple |
| CTF/NF-1 | GCCAAG |  | Simple |
| classic | AGGTCA(.)(.)(.)AGACCA |  | Java |
| PTH | GGTTCA(.)(.)(.)CAGACA |  | Java |
| Insulin | GGGTCA(.)(.)GGGGGCA |  | Java |
| MIS | GGGTGA(.)(.)(.)GGGACA |  | Java |
| osteocalcin | GGGTGA(.)(.)(.)GGGGCA |  | Java |
| CYP24A1p | AGGTGA(.)(.)(.)AGGGCG |  | Java |
| CYP24A1-d | AGTTCA(.)(.)(.)GGTGTG |  | Java |
| IGFBP-3 | GGTTCA(.)(.)(.)GGTGCA |  | Java |
| osteopontin | gggtcg(.)(.)(.)ggttca |  | Java |
| osteoblast | agggca(.)(.)(.)agttcg |  | Java |
| RANKL1 | TGAACT(.)(.)(.)ACAACC |  | Java |
| RANK1-2 | acaact(.)(.)(.)tgactt |  | Java |
| rankl2 | gggtca(.)(.)(.)agggct |  | Java |
| rankl3 | ggttca(.)(.)(.)agttct |  | Java |
| HLA | gggtggagaggggtca |  | Simple |
| CaBP 9K | GGGTGT(.)(.)(.)AAGCCC |  | Java |
| KSR-1 VDRE | GGTGCA(.)(.)(.)AGGTCA |  | Java |
| LRP5-p2 | ggctca(.)(.)(.)gggttc |  | Java |
| HGF VDRE | ccgggaaactggggtcagt |  | Simple |
| RGKTSA | (A\|G)G(G\|T)T(C\|G)A |  | Java |
| RGKTSA1 | (A\|G)G(G\|T)T(C\|G)A(.)(A\|G)G(G\|T)T(C\|G)A |  | Java |
| RGKTSA2 | (A\|G)G(G\|T)T(C\|G)A(.)(.)(A\|G)G(G\|T)T(C\|G)A |  | Java |
| RGKTSA3 | (A\|G)G(G\|T)T(C\|G)A(.)(.)(.)(A\|G)G(G\|T)T(C\|G)A |  | Java |
| RGKTSA4 | (A\|G)G(G\|T)T(C\|G)A(.)(.)(.)(.)(A\|G)G(G\|T)T(C\|G)A |  | Java |
| RGKSTA5 | (A\|G)G(G\|T)T(C\|G)A(.)(.)(.)(.)(.)(A\|G)G(G\|T)T(C\|G)A |  | Java |
| RGKSTA6 | (A\|G)G(G\|T)T(C\|G)A(.)(.)(.)(.)(.)(.)(A\|G)G(G\|T)T(C\|G)A |  | Java |
| RG7 | (A\|G)G(G\|T)T(C\|G)A(.)(.)(.)(.)(.)(.)(.)(A\|G)G(G\|T)T(C\|G)A |  | Java |
| RG8 | (A\|G)G(G\|T)T(C\|G)A(.)(.)(.)(.)(.)(.)(.)(.)(A\|G)G(G\|T)T(C\|G)A |  | Java |
| RG9 | (A\|G)G(G\|T)T(C\|G)A(.)(.)(.)(.)(.)(.)(.)(.)(.)(A\|G)G(G\|T)T(C\|G)A |  | Java |
| RG10 | (A\|G)G(G\|T)T(C\|G)A(.)(.)(.)(.)(.)(.)(.)(.)(.)(.)(A\|G)G(G\|T)T(C\|G)A |  | Java |
| Opposite0 | (A\|G)G(G\|T)T(C\|G)AT(G\|C)A(C\|A)C(T\|C) |  | Java |
| op1 | (A\|G)G(G\|T)T(C\|G)A(.)T(G\|C)A(C\|A)C(T\|C) |  | Java |
| OP2 | (A\|G)G(G\|T)T(C\|G)A(.)(.)T(G\|C)A(C\|A)C(T\|C) |  | Java |
| OP3 | (A\|G)G(G\|T)T(C\|G)A(.)().(.)T(G\|C)A(C\|A)C(T\|C) |  | Java |
| OP4 | (A\|G)G(G\|T)T(C\|G)A(.)(.)(.)(.)T(G\|C)A(C\|A)C(T\|C) |  | Java |
| lrp5 | gggtca(.)(.)(.)tcattc |  | Java |
| OP5 | (A\|G)G(G\|T)T(C\|G)A(.)(.)(.)(.)(.)T(G\|C)A(C\|A)C(T\|C) |  | Java |
| OP6 | (A\|G)G(G\|T)T(C\|G)A(.)(.)(.)(.)(.)(.)T(G\|C)A(C\|A)C(T\|C) |  | Java |
| OP7 | (A\|G)G(G\|T)T(C\|G)A(.)(.)(.)(.)(.)(.)(.)T(G\|C)A(C\|A)C(T\|C) |  | Java |
| Opposite-3 | (A\|G)G(G\|T)T(C\|G)A(C\|A)C(T\|C) |  | Java |
| opositerevers | T(C\|G)A(C\|A)C(T\|C)(A\|G)G(G\|T)T(C\|G)A |  | Java |
| OPrevers1 | T(C\|G)A(C\|A)C(T\|C)(.)(A\|G)G(G\|T)T(C\|G)A |  | Java |
| OPr2 | T(C\|G)A(C\|A)C(T\|C)(.)(.)(A\|G)G(G\|T)T(C\|G)A |  | Java |
| OPr3 | T(C\|G)A(C\|A)C(T\|C)(.)(.)(.)(A\|G)G(G\|T)T(C\|G)A |  | Java |
| OPr4 | T(C\|G)A(C\|A)C(T\|C)(.)(.)(.)(.)(A\|G)G(G\|T)T(C\|G)A |  | Java |
| OPr5 | T(C\|G)A(C\|A)C(T\|C)(.)(.)(.)(.)(.)(A\|G)G(G\|T)T(C\|G)A |  | Java |
| OPr6 | T(C\|G)A(C\|A)C(T\|C)(.)(.)(.)(.)(.)(.)(A\|G)G(G\|T)T(C\|G)A |  | Java |
| GTGACGTCAC | GTGACGTCAC |  | Simple |
| CRE | ttacgtgt |  | Simple |
| CRE1 | aacgtta |  | Simple |
| classic-p2 | AGACCA |  | Simple |
| inslin-p1 | GGGTCA |  | Simple |
| insulin-p2 | GGGGCA |  | Simple |
| mis-p2 | GGGACA |  | Simple |
| osteocalcin-p2 | GGGGCA |  | Simple |
| cyp-p1-1 | AGGTGA |  | Simple |
| cyp2-p2 | GGTGTG |  | Simple |
| igf-p2 | GGTGCA |  | Simple |
| osteopontin-p1 | gggtcg |  | Simple |
| osteopontin-p2 | ggttca |  | Simple |
| ostoblast-p1 | agggca |  | Simple |
| osteo-p2 | agttcg |  | Simple |
| rankl-p1 | TGAACT |  | Simple |
| rankl-p2 | ACAACC |  | Simple |
| lrp1-p1 | ggctca |  | Simple |
| lrp1-p2 | gggttc |  | Simple |
| rankl2-p2 | tcattc |  | Simple |
| RANKL3-p2 | agttct |  | Simple |
| RANKL3-p1 | ggttca |  | Simple |
| CYP2-p1 | AGTTCA |  | Simple |
| CYP-p2 | AGGGCG |  | Simple |
| GGGGCA | GGGGCA |  | Simple |
| thriple | (A\|G)G(G\|T)T(C\|G)A(.)(.)AGTTCGAGACCA |  | Java |
| PUS3 | AGGGCA(.)(.)(.)AGTTCG |  | Java |
| PUS3-1 | GGTTCA(.)(.)(.)AGTTCT |  | Java |
| PUS3-2 | agggca(.)(.)(.)ggggca |  | Java |
| MINPP1 | AGGGCA(.)(.)(.)GGCGGG |  | Java |
| MINPP1-2 | AGGTTA(.)(.)(.)GGGTCA |  | Java |
| PTH-P2 | CAGACA |  | Simple |
| COPB2 | TGAACT(.)(.)(.)AGGTGA |  | Java |
| keap1-1 | AGGCCA(.)(.)(.)GGGGCA |  | Java |
| KEAP1-2 | AGGGCA(.)(.)(.)GGCTCA |  | Java |
| KEAP1-3 | TGAACT(.)(.)(.)GGGCCA |  | Java |
| KEAP1-4 | GGGGGA(.)(.)(.)GGGCGA |  | Java |
| HSPH1-1 | GGGGTA(.)(.)(.)CAGACA |  | Java |
| HSPH1-2 | GGGTCA(.)(.)(.)AGGGCA |  | Java |
| KSR-1/2 | AATGGA(.)(.)(.)CAGACA |  | Java |
| hPTH | TCAACT(.)(.)(.)GGTTCA |  | Java |
| RelB1 | CGGTCA(.)(.)(.)TGGTCT |  | Java |
| RelB2 | GGTTCA(.)(.)(.)CCCACT |  | Java |
| CETN3 | AGGCGA(.)(.)(.)AGGGGA |  | Java |
| CETN3-2 | AGACCA(.)(.)(.)GGGGCA |  | Java |
| HSPH1-3 | AGGCCA(.)(.)(.)GGGGCA |  | Java |
| Y-RNA-1 | GGGTTA(.)(.)(.)AGACCA |  | Java |
| Y-RNA-2 | AGACCA(.)(.)(.)AGGGCA |  | Java |
| Y-RNA-3 | AGGGGA(.)(.)(.)GGCTCA |  | Java |
| Y-RNA-4 | AGAACT(.)(.)(.)TGAACT |  | Java |
| TNNI3K-1 | GGGTCA(.)(.)(.)ACAACC |  | Java |
| TNNI3K-2 | AGGTGA(.)(.)(.)AGGTCA |  | Java |
| TNNI3K-3 | AGGGCG(.)(.)(.)GGGGGA |  | Java |
| regulatory | (A\|G\|C)(A\|G)GG(T\|C\|G)(C\|G\|T)A |  | Java |
| Part1 | (A\|G\|C)(A\|G)G(G\|T\|C)(T\|C\|G)(C\|G\|T)A |  | Java |
| Ram2 | (A\|G\|C)(A\|G)G(G\|T\|C)(T\|C\|G)(C\|G\|T)A(.)(.)(A\|T\|G\|C)(G\|A\|C)(A\|G)G(T\|G)(T\|G\|C)(C\|T)(A\|G\|T\|C) |  | Java |
| hTRPV66 | AGGTCA(.)(.)(.)AGTTCA |  | Java |
| hTRPV66 | GGGTCA(.)(.)(.)GGTTCG |  | Java |
| hTRPV66 | AGGTCA(.)(.)(.)GGTTCA |  | Java |
| hCYP3A41 | GGGTCA(.)(.)(.)AGTTCA |  | Java |
| hp2159 | AGGGAG(.)(.)(.)GGTTCA |  | Java |
| hFOXO160 | GGGTCA(.)(.)(.)AGGTGA |  | Java |
| hWise | AGGACA(.)(.)(.)GGGACA |  | Java |
| ZNF287 | GGGCGA(.)(.)(.)AGGGGA |  | Java |
| PTRH2 | TCATTC(.)(.)(.)AGGGCA |  | Java |
| CETN3- | GGGTTC(.)(.)(.)AGTTCT |  | Java |
| CD83 | GGGCCA(.)(.)(.)GGGTTA |  | Java |
