# Supplementary material for: Influence of Vitamin D Status and Vitamin D3 Supplementation on Genome Wide Expression of White Blood Cells: A Randomized Double-Blind Clinical Trial
Source: PLoS One. 2013 Mar 20;8(3):e58725. doi: 10.1371/journal.pone.0058725 (PMC3604145; doi:10.1371/journal.pone.0058725)
Supplement: Table S3 — The housekeeping genes that used as negative control for VDREs searching. Expression of these housekeeping genes after vitamin D supplementation was not changed. There were no sequences of candidate VDREs in 100 kb upstream of TSS of these housekeeping genes. The list of these housekeeping genes and the region of the study is summarized in the table. (DOCX) [file pone.0058725.s004.docx]

| **Genes symbol and gene ID** | **Description** | **Location** | **Regions for searching** |
| --- | --- | --- | --- |
| **ALDOA ENSG00000149925** | aldolase A, fructose-bisphosphate [Source:HGNC Symbol;Acc:414] | [Chromosome 16: 30,064,411-30,081,778](http://www.ensembl.org/Homo_sapiens/Location/View?db=core;g=ENSG00000149925;r=16:30064411-30081778) forward strand | GRCh37:16:29973917:30100752:1 |
| **PGK1 ENSG00000102144** | phosphoglycerate kinase 1 [Source:HGNC Symbol;Acc:8896] | [Chromosome X: 77,320,685-77,384,793](http://www.ensembl.org/Homo_sapiens/Location/View?db=core;g=ENSG00000102144;r=X:77320685-77384793) forward strand | GRCh37:X:77309073:77450502:1 |
| **RPLP1 ENSG00000137818** | ribosomal protein, large, P1 [Source:HGNC Symbol;Acc:10372] | [Chromosome 15: 69,745,123-69,748,255](http://www.ensembl.org/Homo_sapiens/Location/View?db=core;g=ENSG00000137818;r=15:69745123-69748255) forward strand | GRCh37:15:69641267:69792199:1 |
| **NONO ENSG00000147140** | non-POU domain containing, octamer-binding [Source:HGNC Symbol;Acc:7871] | [Chromosome X: 70,503,042-70,521,018](http://www.ensembl.org/Homo_sapiens/Location/View?db=core;g=ENSG00000147140;r=X:70503042-70521018) forward strand | GRCh37:X:70415282:70683274:1 |
| **RPL19 ENSG00000108298** | ribosomal protein L19 [Source:HGNC Symbol;Acc:10312] | [Chromosome 17: 37,356,536-37,360,980](http://www.ensembl.org/Homo_sapiens/Location/View?db=core;g=ENSG00000108298;r=17:37356536-37360980;t=ENST00000225430) forward strand | GRCh37:17:37274314:37370499:1 |
| **LDHA ENSG00000134333** | lactate dehydrogenase A [Source:HGNC Symbol;Acc:6535] | [Chromosome 11: 18,415,935-18,429,972](http://www.ensembl.org/Homo_sapiens/Location/View?db=core;g=ENSG00000134333;r=11:18415935-18429972) forward strand | GRCh37:11:18313174:18441023:1 |
| **RPS27A ENSG00000143947** | ribosomal protein S27a [Source:HGNC Symbol;Acc:10417] | [Chromosome 2: 55,459,039-55,462,989](http://www.ensembl.org/Homo_sapiens/Location/View?db=core;g=ENSG00000143947;r=2:55459039-55462989) forward strand | GRCh37:2:55422730:55582880:1 |
| **GAPDH ENSG00000111640** | glyceraldehyde-3-phosphate dehydrogenase [Source:HGNC Symbol;Acc:4141] | [Chromosome 12: 6,643,093-6,647,537](http://www.ensembl.org/Homo_sapiens/Location/View?db=core;g=ENSG00000111640;r=12:6643093-6647537) forward strand | GRCh37:12:6593910:6724656:1 |
| **ACTB ENSG00000075624** | actin, beta [Source:HGNC Symbol;Acc:132] | [Chromosome 7: 5,566,782-5,603,415](http://www.ensembl.org/Homo_sapiens/Location/View?db=core;g=ENSG00000075624;r=7:5566782-5603415) reverse strand | GRCh37:7:5543774:5738189:1 |
| **B2M ENSG00000166710** | beta-2-microglobulin [Source:HGNC Symbol;Acc:914] | [Chromosome 15: 45,003,675-45,011,075](http://www.ensembl.org/Homo_sapiens/Location/View?db=core;g=ENSG00000166710;r=15:45003675-45011075) forward strand | GRCh37:15:44968868:45010110:1 |
| **HPRT1 ENSG00000165704** | hypoxanthine phosphoribosyltransferase 1 [Source:HGNC Symbol;Acc:5157 | [Chromosome X: 133,594,183-133,654,543](http://www.ensembl.org/Homo_sapiens/Location/View?db=core;g=ENSG00000165704;r=X:133594183-133654543) forward strand | GRCh37:X:133551470:133705933:1 |
| **RPS9 ENSG00000170889** | ribosomal protein S9 [Source:HGNC Symbol;Acc:10442 | [Chromosome 19: 54,704,610-54,752,862](http://www.ensembl.org/Homo_sapiens/Location/View?db=core;g=ENSG00000170889;r=19:54704610-54752862) forward strand | GRCh37:19:54528888:54724518:1 |
