# Supplementary material for: Influence of Vitamin D Status and Vitamin D3 Supplementation on Genome Wide Expression of White Blood Cells: A Randomized Double-Blind Clinical Trial
Source: PLoS One. 2013 Mar 20;8(3):e58725. doi: 10.1371/journal.pone.0058725 (PMC3604145; doi:10.1371/journal.pone.0058725)
Supplement: Table S4 — The complete list of 291 genes that affected by vitamin D3 supplementation. (DOCX) [file pone.0058725.s005.docx]

| Gene.Symbol | mRNA.Accession |
| --- | --- |
| --- | ENST00000386870 |
| OSM | NM_020530 |
| EGR1 | NM_001964 |
| NFIL3 | NM_005384 |
| JUNB | NM_002229 |
| --- | ENST00000411180 |
| H3F3B | NM_005324 |
| JUN | NM_002228 |
| JUND | NM_005354 |
| NR4A2 | NM_006186 |
| C17orf91 | NM_032895 |
| CCNJL | NM_024565 |
| PIM3 | NM_001001852 |
| AXUD1 | NM_033027 |
| IER2 | NM_004907 |
| TNNI3K | NM_015978 |
| ZFP36 | NM_003407 |
| --- | ENST00000365538 |
| SIK1 | NM_173354 |
| PTP4A1 | NM_003463 |
| KLF11 | NM_003597 |
| KLF10 | NM_005655 |
| CD83 | NM_004233 |
| --- | ENST00000411154 |
| TMCC3 | NM_020698 |
| LOC100132005 | AK092662 |
| PHLPP | NM_194449 |
| --- | AF318321 |
| SNORA38 | NR_002971 |
| --- | ENST00000364900 |
| --- | ENST00000383975 |
| RNU1A | NR_004421 |
| RNU2-1 | NR_002716 |
| FGF5 | NM_004464 |
| THAP10 | NM_020147 |
| --- | ENST00000408224 |
| METRNL | NM_001004431 |
| --- | ENST00000386838 |
| KIAA1210 | NM_020721 |
| C18orf16 | AK055069 |
| NKAIN1 | NM_024522 |
| --- | ENST00000385490 |
| PXDNL | NM_144651 |
| C21orf119 | BC007928 |
| --- | ENST00000364716 |
| --- | ENST00000410926 |
| --- | ENST00000364233 |
| --- | ENST00000383864 |
| SNORA48 | NR_002918 |
| SNORA3 | NR_002580 |
| TNFAIP3 | NM_006290 |
| NFKBIA | NM_020529 |
| ZFAND2A | NM_182491 |
| --- | ENST00000386508 |
| SNORD59B | NR_003046 |
| HIST1H1E | NM_005321 |
| --- | ENST00000410897 |
| YRDC | NM_024640 |
| --- | ENST00000383924 |
| SNORA75 | NR_002921 |
| RNF139 | NM_007218 |
| SNORA67 | NR_002912 |
| SNORA9 | NR_002952 |
| LOC441150 | NM_001008739 |
| SBDS | NM_016038 |
| --- | ENST00000363367 |
| --- | ENST00000386378 |
| ARRDC3 | NM_020801 |
| SNORA27 | NR_002575 |
| GRASP | NM_181711 |
| IRS2 | NM_003749 |
| --- | ENST00000365541 |
| H1FX | NM_006026 |
| --- | ENST00000391093 |
| UNQ5814 | AY358167 |
| --- | ENST00000385756 |
| ATF4 | NM_001675 |
| --- | ENST00000384074 |
| SNORA69 | NR_002584 |
| --- | ENST00000387180 |
| --- | ENST00000364774 |
| --- | ENST00000386782 |
| --- | ENST00000387655 |
| MIS12 | NM_024039 |
| RASA1 | NM_002890 |
| TGDS | NM_014305 |
| DTWD1 | NM_020234 |
| SNRPN // SNORD115-40 | NR_003355 |
| --- | ENST00000384378 |
| --- | ENST00000386019 |
| LOC100128868 | AY143171 |
| RWDD3 | NM_015485 |
| --- | ENST00000387732 |
| C6orf182 | NM_001083535 |
| PDE4DIP | AB042555 |
| --- | ENST00000385451 |
| C10orf2 | NM_021830 |
| --- | ENST00000363110 |
| PDP2 | NM_020786 |
| ZNF225 | NM_013362 |
| RBBP5 | NM_005057 |
| ZNF607 | NM_032689 |
| --- | ENST00000364083 |
| GNRH1 | NM_000825 |
| LOC440957 | NM_001124767 |
| --- | ENST00000387056 |
| --- | ENST00000362376 |
| C5orf34 | BC036867 |
| GGCX | NM_000821 |
| --- | ENST00000385762 |
| --- | ENST00000363300 |
| TAS2R4 | NM_016944 |
| RBM12B | NM_203390 |
| --- | ENST00000385952 |
| TAS2R3 | NM_016943 |
| LOC492311 | NM_001007189 |
| --- | AY831680 |
| --- | ENST00000363668 |
| ZNF287 | NM_020653 |
| ORC2L | NM_006190 |
| TUBD1 | NM_016261 |
| CCDC76 | NM_019083 |
| ZNF780B | NM_001005851 |
| RFXAP | NM_000538 |
| PCID2 | BC008975 |
| ZNF616 | BC032805 |
| --- | ENST00000363399 |
| HELB | NM_033647 |
| C11orf61 | NM_024631 |
| ZNF397 | NM_001135178 |
| LOC100131860 | AK097109 |
| ZNF284 | NM_001037813 |
| --- | ENST00000385919 |
| C12orf11 | NM_018164 |
| STK38 | NM_007271 |
| --- | BC012036 |
| --- | AF074983 |
| N6AMT1 | NM_013240 |
| ZFP62 | NM_152283 |
| METTL4 | NM_022840 |
| CXCR7 | NM_020311 |
| RIC8B | NM_018157 |
| HOMEZ | NM_020834 |
| C2orf42 | BC005079 |
| CEP76 | NM_024899 |
| CUZD1 | NM_022034 |
| LOC653354 | ENST00000372828 |
| KIAA1009 | NM_014895 |
| --- | ENST00000342007 |
| ZNF691 | NM_015911 |
| PTRH2 | NM_016077 |
| LOC100128751 | AY194294 |
| C11orf54 | BC012298 |
| XIAP | NM_001167 |
| GART | NM_000819 |
| POLA2 | NM_002689 |
| KEAP1 | NM_203500 |
| STIP1 | NM_006819 |
| ZNF701 | NM_018260 |
| BUD13 | NM_032725 |
| SLC39A7 | NM_006979 |
| FXR2 | NM_004860 |
| FAM118B | NM_024556 |
| TNFAIP8L2 | NM_024575 |
| NDUFAF1 | NM_016013 |
| COPB2 | NM_004766 |
| C8orf41 | NM_025115 |
| --- | ENST00000385991 |
| LOC646938 | BC141940 |
| SOS1 | NM_005633 |
| GTF2E1 | NM_005513 |
| HSPA4 | NM_002154 |
| HSPH1 | NM_006644 |
| CCDC117 | NM_173510 |
| INTS7 | NM_015434 |
| C21orf91 | NM_001100420 |
| TRIM27 | NM_006510 |
| PPID | NM_005038 |
| KRCC1 | NM_016618 |
| RNASEL | NM_021133 |
| ZNF232 | NM_014519 |
| ZNF473 | NM_015428 |
| POP1 | NM_015029 |
| TAF1A | NM_005681 |
| MTO1 | NM_133645 |
| NAPEPLD | NM_001122838 |
| IWS1 | NM_017969 |
| ZNF587 | AF294842 |
| ZDHHC16 | NM_198046 |
| MIZF | NM_015517 |
| KIAA0859 | NM_015935 |
| C1orf25 | NM_030934 |
| LARP4 | NM_052879 |
| FAM98A | NM_015475 |
| ZNF223 | NM_013361 |
| GIMAP2 | NM_015660 |
| KIAA1279 | NM_015634 |
| ZNF175 | NM_007147 |
| THAP2 | NM_031435 |
| MED7 | NM_004270 |
| NUP43 | NM_198887 |
| GVIN1 | NR_003945 |
| BBS7 | NM_176824 |
| GIMAP6 | NM_024711 |
| GIMAP1 | NM_130759 |
| GIMAP7 | NM_153236 |
| --- | ENST00000385695 |
| ZNF320 | NM_207333 |
| BTN3A2 | NM_007047 |
| --- | ENST00000385628 |
| FLJ21272 | AK024925 |
| --- | ENST00000410767 |
| CEACAM21 | NM_001098506 |
| RCCD1 | NM_033544 |
| UTP3 | NM_020368 |
| PUS3 | NM_031307 |
| MFAP1 | NM_005926 |
| ZNF137 | NR_023311 |
| ZNF17 | NM_006959 |
| C11orf73 | NM_016401 |
| ARSK | NM_198150 |
| ZNF45 | NM_003425 |
| MRPL50 | NM_019051 |
| NUP107 | NM_020401 |
| ZFP3 | NM_153018 |
| KIAA0406 | BC013121 |
| ZNF283 | NM_181845 |
| SLC30A6 | NM_017964 |
| GEMIN6 | NM_024775 |
| --- | BC019340 |
| CETN3 | NM_004365 |
| ORAOV1 | NM_153451 |
| NOC3L | NM_022451 |
| SUV420H1 | NM_017635 |
| CEP110 | NM_007018 |
| GLS | NM_014905 |
| NHLRC2 | NM_198514 |
| C8orf44 | BC014448 |
| ALKBH1 | NM_006020 |
| SLC30A5 | NM_022902 |
| ZNF852 | AK296954 |
| SNORA46 | NR_002978 |
| CHORDC1 | NM_012124 |
| ZCCHC8 | NM_017612 |
| MED17 | NM_004268 |
| ZNF235 | NM_004234 |
| ZNF780A | NM_001010880 |
| RBM5 | NM_005778 |
| TMEM68 | NM_152417 |
| INSIG2 | NM_016133 |
| ZNF322A | NM_024639 |
| PGBD2 | NM_170725 |
| GGPS1 | NM_004837 |
| GALK2 | NM_001001556 |
| GOPC | NM_020399 |
| ZNRD1 | NM_170783 |
| SLC35B3 | NM_015948 |
| C18orf54 | NM_173529 |
| TMEM184C | NM_018241 |
| HCFC2 | NM_013320 |
| SNAPC3 | NM_001039697 |
| MKKS | NM_018848 |
| C5orf51 | NM_175921 |
| FPGT | NM_003838 |
| BET1 | NM_005868 |
| MINPP1 | NM_004897 |
| USO1 | NM_003715 |
| HMGCL | NM_000191 |
| GTF2H1 | NM_005316 |
| YIPF4 | NM_032312 |
| --- | ENST00000363371 |
| TRIM13 | NM_213590 |
| HSD17B7P2 | NR_003086 |
| HSD17B7 | NM_016371 |
| C7orf38 | NM_145111 |
| BPNT1 | NM_006085 |
| PIGM | NM_145167 |
| TIA1 | NM_022173 |
| TTC9C | NM_173810 |
| GCET2 | NM_001008756 |
| TRAPPC6B | NM_001079537 |
| SACM1L | NM_014016 |
| NUDCD1 | NM_001128211 |
| BBS10 | NM_024685 |
| TRIP11 | NM_004239 |
| CSTF3 | NM_001326 |
| JRKL | NM_003772 |
| ZNF234 | NM_006630 |
| C17orf80 | NM_017941 |
| POLH | NM_006502 |
| ZNF260 | NM_001012756 |
