# Supplementary material for: Influence of Vitamin D Status and Vitamin D3 Supplementation on Genome Wide Expression of White Blood Cells: A Randomized Double-Blind Clinical Trial
Source: PLoS One. 2013 Mar 20;8(3):e58725. doi: 10.1371/journal.pone.0058725 (PMC3604145; doi:10.1371/journal.pone.0058725)
Supplement: Table S5 — The pathways enriched with down regulated genes after treatment (p<0.05). (DOCX) [file pone.0058725.s006.docx]

| CD40/CD40L signaling |
| --- |
| PI3K/AKT signalling |
| Neurotrophin signaling pathway |
| Downstream signaling in naÔve CD8+ T cells |
| Cd40l signaling pathway |
| Signalling by NGF |
| Erythropoietin mediated neuroprotection through nf-kb |
| Calcineurin-regulated NFAT-dependent transcription in lymphocytes |
| Tnfr2 signaling pathway |
| Cadmium induces dna synthesis and proliferation in macrophages |
| Atm signaling pathway |
| TRAF6 Mediated Induction of the antiviral cytokine IFN-alpha/beta cascade |
| TRKA signalling from the plasma membrane |
| Toll Like Receptor 3 (TLR3) Cascade |
| Canonical NF-kappaB pathway |
| Signaling events mediated by PRL |
| Calcium signaling in the CD4+ TCR pathway |
| ErbB1 downstream signaling |
| Osteopontin-mediated events |
| TNFR1 signaling pathway |
| Role of egf receptor transactivation by gpcrs in cardiac hypertrophy |
| Toll-like receptor pathway |
| Signal transduction through il1r |
| MAPK signaling pathway |
| Signaling events regulated by Ret tyrosine kinase |
| Regulation of target gene expression by AP-1 |
| Mechanism of gene regulation by peroxisome proliferators via ppara |
| IL6-mediated signaling events |
| ATF-2 transcription factor network |
| TGFBR |
| Toll Receptor Cascades |
| Regulation of Androgen receptor activity |
| Keratinocyte differentiation |
| T cell receptor signaling pathway |
| IL2-mediated signaling events |
| Adipocytokine signaling pathway |
| PERK regulated gene expression |
| Epithelial cell signaling in Helicobacter pylori infection |
| AKT phosphorylates targets in the nucleus |
| Acetylation and deacetylation of rela in nucleus |
| Fosb gene expression and drug abuse |
| Gene expression of IL2 by AP-1 |
| IRS activation |
| Negative regulation of the PI3K/AKT network |
| EGFR1 |
| NOD-like receptor signaling pathway |
| BCR signaling pathway |
| LPA receptor mediated events |
| CDC42 signaling events |
| D4gdi signaling pathway |
| B cell receptor signaling pathway |
| Glucocorticoid receptor regulatory network |
| Tsp-1 induced apoptosis in microvascular endothelial cell |
| T cell receptor signaling pathway |
| IKK-NF-kappaB cascade |
| Prostate cancer |
| Pertussis toxin-insensitive ccr5 signaling in macrophage |
| Toll-like receptor signaling pathway |
| Apoptotic dna-fragmentation and tissue homeostasis |
| IKK-NFkB cascade |
| Signal attenuation |
| Viral dsRNA:TLR3:TRIF Complex Activates RIP1 |
| Unfolded Protein Response |
| Innate Immunity Signaling |
| GnRH signaling pathway |
| IL9 |
| NF-kB signaling |
| JNK signaling in the CD4+ TCR pathway |
| SOS-mediated signalling |
| The 41bb-dependent immune response |
| T cell receptor signaling pathway |
| Atypical NF-kappaB pathway |
| Double stranded rna induced gene expression |
| Phosphorylation of mek1 by cdk5/p35 down regulates the map kinase pathway |
| Calcium signaling by hbx of hepatitis b virus |
| Il12 and stat4 dependent signaling pathway in th1 development |
| Repression of pain sensation by the transcriptional regulator dream |
| Bone remodeling |
| Hypoxia-inducible factor in the cardivascular system |
| IL-7 |
| Chaperones modulate interferon signaling pathway |
| NF-kB is activated and signals survival |
| Nerve growth factor pathway (ngf) |
| Akt signaling pathway |
| Mets affect on macrophage differentiation |
| Oxidative stress induced gene expression via nrf2 |
| JNK cascade |
|  |
