# Supplementary material for: Influence of Vitamin D Status and Vitamin D3 Supplementation on Genome Wide Expression of White Blood Cells: A Randomized Double-Blind Clinical Trial
Source: PLoS One. 2013 Mar 20;8(3):e58725. doi: 10.1371/journal.pone.0058725 (PMC3604145; doi:10.1371/journal.pone.0058725)
Supplement: Table S6 — Pathways enriched with up regulated genes after treatment (p<0.05). (DOCX) [file pone.0058725.s007.docx]

| Zinc transporters |
| --- |
| Metal ion SLC transporters |
| Zinc efflux and compartmentalization by the SLC30 family |
| Late Phase of HIV Life Cycle |
| Cell Cycle, Mitotic |
| Transcription |
| Gene Expression |
| HIV Life Cycle |
| Metabolism of non-coding RNA |
| SnRNP Assembly |
| Translesion synthesis by Pol eta |
| Egf signaling pathway |
| Igf-1 signaling pathway |
| Sprouty regulation of tyrosine kinase signals |
| RNA Polymerase I Chain Elongation |
| RNA Polymerase I Promoter Escape |
| RNA Polymerase I Transcription Termination |
| Tpo signaling pathway |
| Down-stream signal transduction |
| RNA Polymerase I Transcription Initiation |
| Generic Transcription Pathway |
| Pdgf signaling pathway |
| EGF receptor (ErbB1) signaling pathway |
| Processing of Capped Intron-Containing Pre-mRNA |
| Activation of the pre-replicative complex |
| NEP/NS2 Interacts with the Cellular Export Machinery |
| Negative Regulation of Glucokinase by Glucokinase Regulatory Protein |
| Nuclear import of Rev protein |
| Signaling by PDGF |
| Transport of Ribonucleoproteins into the Host Nucleus |
| Vpr-mediated nuclear import of PICs |
| E2F mediated regulation of DNA replication |
| Export of Viral Ribonucleoproteins from Nucleus |
| Rev-mediated nuclear export of HIV-1 RNA |
| Mitotic Prometaphase |
| Interactions of Rev with host cellular proteins |
| Interactions of Vpr with host cellular proteins |
| Transport of the SLBP independent Mature mRNA |
| DNA Damage Bypass |
| Formation of Acetoacetic Acid |
| Translesion synthesis by DNA polymerases bypassing lesion on DNA template |
| Transport of the SLBP Dependant Mature mRNA |
| Formation and Maturation of mRNA Transcript |
| M Phase |
| Basal transcription factors |
| Transport of Mature mRNA Derived from an Intronless Transcript |
| Glucose uptake |
| Transport of Mature mRNAs Derived from Intronless Transcripts |
| RNA Polymerase II Transcription |
| D-Glutamine and D-glutamate metabolism |
| Synthesis of Ketone Bodies |
| Post-translational protein modification |
| Signaling events regulated by Ret tyrosine kinase |
| RNA Polymerase I Promoter Clearance |
| HIV-1 Transcription Initiation |
| HIV-1 Transcription Pre-Initiation |
| RNA Polymerase II HIV-1 Promoter Escape |
| RNA Polymerase II Promoter Escape |
| RNA Polymerase II Transcription Initiation |
| RNA Polymerase II Transcription Initiation And Promoter Clearance |
| RNA Polymerase II Transcription Pre-Initiation |
| Ketone body metabolism |
| SMAC binds to IAPs |
| SMAC-mediated apoptotic response |
| SMAC-mediated dissociation of IAP:caspase complexes |
| Insulin Pathway |
| RNA Polymerase I Transcription |
| Amino sugar and nucleotide sugar metabolism |
| HIV Infection |
| Assembly of the ORC complex at the origin of replication |
| DNA replication initiation |
| De novo synthesis of IMP |
| Telomere C-strand synthesis initiation |
| Taste transduction |
| IL4 |
| TGF-beta receptor signaling |
| Apoptotic factor-mediated response |
| Aspartate, asparagine, glutamate, and glutamine metabolism |
| Inhibition of replication initiation of damaged DNA by Rb/E2F1 |
| Transport of Mature mRNA derived from an Intron-Containing Transcript |
| Signaling events activated by Hepatocyte Growth Factor Receptor (c-Met) |
