# Supplementary material for: Influence of Vitamin D Status and Vitamin D3 Supplementation on Genome Wide Expression of White Blood Cells: A Randomized Double-Blind Clinical Trial
Source: PLoS One. 2013 Mar 20;8(3):e58725. doi: 10.1371/journal.pone.0058725 (PMC3604145; doi:10.1371/journal.pone.0058725)
Supplement: Protocol S1 — Trial Protocol. (PDF) [file pone.0058725.s009.pdf]

## 1.0 General Information

### 1.1 *Please enter the full title of your study (Spell out acronyms):

THE EFFECT OF VITAMIN D ON MEASURES OF BONE HEALTH AND GENE EXPRESSION

### 1.2 *Please enter the Study Nickname you would like to use to reference the study:

28351

## 2.0 Add Department(s)

### 2.1 List of Departments associated with this study:

| Primary Dept? | Department Name |
|---|---|
| (selected) | **BU** - *MED* - ENDOCRINE LABORATORY |

## 3.0 Assign key study personnel(KSP) access to the study

### 3.1 *Please add a Principal Investigator for the study:

Holick, Michael

Select if applicable

- [ ] Student
- [ ] Resident
- [ ] Fellow

If the Principal Investigator is a Student, Resident, or Fellow, the name of the Faculty Advisor must be supplied in Section 3.4 below.

### 3.2 If applicable, please select the Protocol Staff personnel:

A) Additional Investigators

Chen, Tai
Co-Investigator
Spira, Avrum
Co-Investigator

B) Research Support Staff

No Research Staff have been added.

**3.3** ***Please add a Study Contact:**

1. Biancuzzo, Rachael

---

The Study Contact(s) will receive all important system notifications along with the Principal Investigator. (e.g. The study contact(s) are typically either the Study Coordinator or the Principal Investigator themselves).

**3.4** **If applicable, please add a Faculty Advisor:**

No Faculty Advisors have been added.

**3.5** **If applicable, please select the Designated Department Approval(s):**

1. Antman, Karen
   Dean Medical School
2. Bhasin, Shalender, MD
   Department Chair
3. Hong, Hyeseon
   Pharmacy
4. Roth, Mary-Tara, RN, MSN, MPH
   GCRC
5. Xiarhos, Elizabeth
   Institutional Biosafety Committee

**3.6** **If applicable, please select the Administrative Assistant(s)**

No Administrative Assistants have been added.

List here anyone performing administrative tasks only (not engaged in research and having no contact with subjects or identifiable data; where certification/recertification and COI disclosure form are not required)- Click on (?) icon for more info.

## 4.0 External non-BU/BMC Investigators

**4.1** **In this section, only list non-BU/BMC investigators (not a full-time or permanent part-time employee of BMC, BU, BPHC, etc.). Any BU/BMC personnel should be listed in the KSP section (3rd section)**

List here all non-BU/BMC persons working on the protocol who will be *engaged in the research on behalf of BU/BMC*. This includes all persons who are conducting research *under an Authorization Agreement (IAA) with BU/BMC IRB*.

No External Personnel have been associated.

**4.2 Does this study involve participation of non-BUMC investigators who are determined to be "not-engaged" in the research?**

○ Yes ○ No

**If you answered Yes above, indicate in the text box below; the names of the non-BUMC investigators, all study activities they will be performing, the names of their institutions, and why they are determined to be NOT-Engaged in the research (based on the OHRP engagement guidance).**

**4.3 Study Attachments**

Click on the link below to attach any necessary documents related to external non-BU/BMC personnel.

No electronic document has been associated.

## 5.0 Investigator Information from INSPIR I

**5.1 This section had been migrated from INSPIR I.**
**- If this is a new study, please skip this section (click Save and Continue).**
**- If this is a study that was migrated from INSPIR I, DO NOT ADD ANY MORE INVESTIGATORS IN THIS SECTION. YOU CAN ONLY DELETE INVESTIGATORS HERE. All BU/BMC personnel should be listed in the KSP section (3rd section), and all non-BU/BMC investigators should be listed in the External non-BU/BMC Investigators section (4rth section).**

**KSP Info**

**Name:**

If you do not see the Key Study Personnel in the dropdown list above, enter it here:

JOSEPH LYTLE

**Department:**

**Location / Address:**

**Phone:**

617-638-8860

**Fax:**

**Email:**

joe.lytle@bmc.org

**Role in Study (ie. Study Coordinator, Statistician, Study Nurse, MD, etc):**

research assistant

A project specific Conflict of Interest Disclosure Form might be required for this person, to be submitted to the appropriate office.

**Additional Personnel Info**

**Is this person an employee of BU, BMC, Boston Public Health Commission (BPHC) or a Boston Healthnet Community Health Center (CHC)?**

○ Yes ◉ No

**For non-BUMC (non-BU/non-BMC) researchers ONLY, please indicate:**

**1. His/Her role in this study**
**2. Who will supervise his/her conduct in this study**
**3. Degrees achieved**
**4. Licenses held (e.g. MD, RN, RPh, LICSW, DMD/DDS, DO, other)**
**5. Professional liability insurance coverage (type and coverage limits)**
**6. University and/or hospital affiliations relevant to this study**
**7. Whether this researcher is considered "engaged" at his/her institution according to the OHRP Guidance.**

1. Joseph is an undergraduate student at BU.

**Did this person identify a conflict of interest in this form?**

○ Yes ◉ No

He will help the graduate students in recruiting subjects.
2. Dr. Holick will supervise him conduct in this study.
3. None. He is currently working on getting his B.S. at Boston University.
4. None
5. N/A
6. N/A
7. He is "engaged" at BU.

**Name:**

If you do not see the Key Study Personnel in the dropdown list above, enter it here:

ESTHER KIM

**Department:**

**Location / Address:**

**Phone:**

617-638-8860

**Fax:**

617-638-8882

**Email:**

estherinaekim@gmail.com

**Role in Study (ie. Study Coordinator, Statistician, Study Nurse, MD, etc):**

research assistant

A project specific Conflict of Interest Disclosure Form might be required for this person, to be submitted to the appropriate office.

**Did this person identify a conflict of interest in this form?**

○ Yes ◉ No

**Is this person an employee of BU, BMC, Boston Public Health Commission (BPHC) or a Boston Healthnet Community Health Center (CHC)?**

○ Yes ◉ No

**For non-BUMC (non-BU/non-BMC) researchers ONLY, please indicate:**

**1. His/Her role in this study**
**2. Who will supervise his/her conduct in this study**
**3. Degrees achieved**
**4. Licenses held (e.g. MD, RN, RPh, LICSW, DMD/DDS, DO, other)**
**5. Professional liability insurance coverage (type and coverage limits)**
**6. University and/or hospital affiliations relevant to this study**
**7. Whether this researcher is considered "engaged" at his/her institution according to the OHRP Guidance.**

1. Esther is a master's student at BUSM. She will recruit, enroll and consent subjects, conduct study visits.
2. Dr. Holick will supervise her conduct in this study.
3. B.S.
4. None
5. N/A
6. N/A
7. She is "engaged" at BUSM.

**Name:**

If you do not see the Key Study Personnel in the dropdown list above, enter it here:

HATAIKARN NIMITPHONG

**Department:**

**Is this person an employee of BU, BMC, Boston Public Health Commission (BPHC) or a Boston Healthnet Community Health Center**

**Location / Address:**

85 East Newton Street

**Phone:**

**Fax:**

617.638.8882

**Email:**

doctorpo@bu.edu

**Role in Study (ie. Study Coordinator, Statistician, Study Nurse, MD, etc):**

co-investigator

A project specific Conflict of Interest Disclosure Form might be required for this person, to be submitted to the appropriate office.

**Did this person identify a conflict of interest in this form?**

○ Yes ● No

**(CHC)?**

● Yes ○ No

**For non-BUMC (non-BU/non-BMC) researchers ONLY, please indicate:**

**1. His/Her role in this study**
**2. Who will supervise his/her conduct in this study**
**3. Degrees achieved**
**4. Licenses held (e.g. MD, RN, RPh, LICSW, DMD/DDS, DO, other)**
**5. Professional liability insurance coverage (type and coverage limits)**
**6. University and/or hospital affiliations relevant to this study**
**7. Whether this researcher is considered "engaged" at his/her institution according to the OHRP Guidance.**

**Name:**

If you do not see the Key Study Personnel in the dropdown list above, enter it here:

EMILY DEMETRIOU

**Department:**

**Location / Address:**

**Phone:**

**Fax:**

**Email:**

emilywood.demetriou@bu.edu

**Role in Study (ie. Study Coordinator, Statistician, Study Nurse, MD, etc):**

co-investigator, MD

A project specific Conflict of Interest Disclosure Form might be required for this person, to be submitted to the appropriate office.

**Did this person identify a conflict of interest in this form?**

**Is this person an employee of BU, BMC, Boston Public Health Commission (BPHC) or a Boston Healthnet Community Health Center (CHC)?**

● Yes ○ No

**For non-BUMC (non-BU/non-BMC) researchers ONLY, please indicate:**

**1. His/Her role in this study**
**2. Who will supervise his/her conduct in this study**
**3. Degrees achieved**
**4. Licenses held (e.g. MD, RN, RPh, LICSW, DMD/DDS, DO, other)**
**5. Professional liability insurance coverage (type and coverage limits)**
**6. University and/or hospital affiliations relevant to this study**
**7. Whether this researcher is considered "engaged" at his/her institution according to the OHRP Guidance.**

○ Yes ◉ No

**Name:**

If you do not see the Key Study Personnel in the dropdown list above, enter it here:

CASSANDRA VOLTAIRE

**Department:**

**Location / Address:**

**Phone:**

857.236.9015

**Fax:**

617.638.8882

**Email:**

cvoltair@bu.edu

**Role in Study (ie. Study Coordinator, Statistician, Study Nurse, MD, etc):**

research assistant

A project specific Conflict of Interest Disclosure Form might be required for this person, to be submitted to the appropriate office.

**Did this person identify a conflict of interest in this form?**

○ Yes ◉ No

**Is this person an employee of BU, BMC, Boston Public Health Commission (BPHC) or a Boston Healthnet Community Health Center (CHC)?**

○ Yes ◉ No

**For non-BUMC (non-BU/non-BMC) researchers ONLY, please indicate:**
**1. His/Her role in this study**
**2. Who will supervise his/her conduct in this study**
**3. Degrees achieved**
**4. Licenses held (e.g. MD, RN, RPh, LICSW, DMD/DDS, DO, other)**
**5. Professional liability insurance coverage (type and coverage limits)**
**6. University and/or hospital affiliations relevant to this study**
**7. Whether this researcher is considered "engaged" at his/her institution according to the OHRP Guidance.**

1. Cassandra is a master's student at BUSM. She will recruit, enroll and consent subjects, conduct study visits.
2. Dr. Holick will supervise her conduct in this study.
3. B.S.
4. None
5. N/A
6. N/A
7. She is "engaged" at BUSM.

**Name:**

If you do not see the Key Study Personnel in the dropdown list above, enter it here:

LENORE DACUYCUY

**Department:**

**Location / Address:**

**Phone:**

408.314.4751

**Fax:**

**Is this person an employee of BU, BMC, Boston Public Health Commission (BPHC) or a Boston Healthnet Community Health Center (CHC)?**

○ Yes ◉ No

**For non-BUMC (non-BU/non-BMC) researchers ONLY, please indicate:**
**1. His/Her role in this study**
**2. Who will supervise his/her conduct in this study**
**3. Degrees achieved**
**4. Licenses held (e.g. MD, RN,**

617.638.8882

**Email:**

dacuycuy@bu.edu

**Role in Study (ie. Study Coordinator, Statistician, Study Nurse, MD, etc):**

research assistant

A project specific Conflict of Interest Disclosure Form might be required for this person, to be submitted to the appropriate office.

**Did this person identify a conflict of interest in this form?**

○ Yes ◉ No

**RPh, LICSW, DMD/DDS, DO, other)**
**5. Professional liability insurance coverage (type and coverage limits)**
**6. University and/or hospital affiliations relevant to this study**
**7. Whether this researcher is considered "engaged" at his/her institution according to the OHRP Guidance.**

1. recruiting and enrolling subjects, conducting study visits.
2. Dr. Michael Holick will supervise her conduct in this study
3. B.A.
4. None
5. N/A
6. N/A
7. Yes, this researcher is "engaged" at BUSM.

**Name:**

If you do not see the Key Study Personnel in the dropdown list above, enter it here:

MICHAEL HOLICK

**Department:**

ENDOCRINE LABORATORY

**Location / Address:**

85 East Newton Street, M-1013

**Phone:**

(617) 638-4545

**Fax:**

(617) 638-8882

**Email:**

MFHOLICK@BU.EDU

**Role in Study (ie. Study Coordinator, Statistician, Study Nurse, MD, etc):**

A project specific Conflict of Interest Disclosure Form might be required for this person, to be submitted to the appropriate office.

**Did this person identify a conflict of interest in this form?**

○ Yes ◉ No

**Is this person an employee of BU, BMC, Boston Public Health Commission (BPHC) or a Boston Healthnet Community Health Center (CHC)?**

◉ Yes ○ No

**For non-BUMC (non-BU/non-BMC) researchers ONLY, please indicate:**
**1. His/Her role in this study**
**2. Who will supervise his/her conduct in this study**
**3. Degrees achieved**
**4. Licenses held (e.g. MD, RN, RPh, LICSW, DMD/DDS, DO, other)**
**5. Professional liability insurance coverage (type and coverage limits)**
**6. University and/or hospital affiliations relevant to this study**
**7. Whether this researcher is considered "engaged" at his/her institution according to the OHRP Guidance.**

**Name:**
If you do not see the Key Study Personnel in the dropdown list above, enter it here:
TAI CHEN

**Department:**
ENDOCRINE LABORATORY

**Location / Address:**

**Phone:**
(617) 638-4543

**Fax:**
[FAX]

**Email:**
TAICHEN@BU.EDU

**Role in Study (ie. Study Coordinator, Statistician, Study Nurse, MD, etc):**
co-investigator

A project specific Conflict of Interest Disclosure Form might be required for this person, to be submitted to the appropriate office.

**Did this person identify a conflict of interest in this form?**

○ Yes ◉ No

**Is this person an employee of BU, BMC, Boston Public Health Commission (BPHC) or a Boston Healthnet Community Health Center (CHC)?**
◉ Yes ○ No

**For non-BUMC (non-BU/non-BMC) researchers ONLY, please indicate:**
**1. His/Her role in this study**
**2. Who will supervise his/her conduct in this study**
**3. Degrees achieved**
**4. Licenses held (e.g. MD, RN, RPh, LICSW, DMD/DDS, DO, other)**
**5. Professional liability insurance coverage (type and coverage limits)**
**6. University and/or hospital affiliations relevant to this study**
**7. Whether this researcher is considered "engaged" at his/her institution according to the OHRP Guidance.**

**Name:**
If you do not see the Key Study Personnel in the dropdown list above, enter it here:
AVRUM SPIRA

**Department:**
PULMONARY CENTER

**Location / Address:**

**Phone:**
638-4860

**Fax:**
[FAX]

**Email:**
ASPIRA@BU.EDU

**Role in Study (ie. Study Coordinator,**

**Is this person an employee of BU, BMC, Boston Public Health Commission (BPHC) or a Boston Healthnet Community Health Center (CHC)?**
◉ Yes ○ No

**For non-BUMC (non-BU/non-BMC) researchers ONLY, please indicate:**
**1. His/Her role in this study**
**2. Who will supervise his/her conduct in this study**
**3. Degrees achieved**
**4. Licenses held (e.g. MD, RN, RPh, LICSW, DMD/DDS, DO, other)**
**5. Professional liability insurance**

**Statistician, Study Nurse, MD, etc):**

co-investigator

A project specific Conflict of Interest Disclosure Form might be required for this person, to be submitted to the appropriate office.

**Did this person identify a conflict of interest in this form?**

○ Yes ◉ No

**Name:**

If you do not see the Key Study Personnel in the dropdown list above, enter it here:

TIMOTHY CALVERT

**Department:**

**Location / Address:**

**Phone:**

617.638.8860

**Fax:**

617.638.8882

**Email:**

nomar@bu.edu

**Role in Study (ie. Study Coordinator, Statistician, Study Nurse, MD, etc):**

research assistant

A project specific Conflict of Interest Disclosure Form might be required for this person, to be submitted to the appropriate office.

**Did this person identify a conflict of interest in this form?**

○ Yes ◉ No

**Name:**

If you do not see the Key Study Personnel in the dropdown list above, enter it here:

**coverage (type and coverage limits)**
**6. University and/or hospital affiliations relevant to this study**
**7. Whether this researcher is considered "engaged" at his/her institution according to the OHRP Guidance.**

**Is this person an employee of BU, BMC, Boston Public Health Commission (BPHC) or a Boston Healthnet Community Health Center (CHC)?**

○ Yes ◉ No

**For non-BUMC (non-BU/non-BMC) researchers ONLY, please indicate:**
**1. His/Her role in this study**
**2. Who will supervise his/her conduct in this study**
**3. Degrees achieved**
**4. Licenses held (e.g. MD, RN, RPh, LICSW, DMD/DDS, DO, other)**
**5. Professional liability insurance coverage (type and coverage limits)**
**6. University and/or hospital affiliations relevant to this study**
**7. Whether this researcher is considered "engaged" at his/her institution according to the OHRP Guidance.**

1. Tim is a master's student at BUSM. He will recruit, enroll and consent subjects, conduct study visits.
2. Dr. Holick will supervise him conduct in this study.
3. B.S.
4. None
5. N/A
6. N/A
7. He is "engaged" at BUSM.

**Is this person an employee of BU,**

RACHAEL BIANCUZZO

**Department:**

**Location / Address:**

**Phone:**

(617) 638-8860

**Fax:**

(617) 638-8882

**Email:**

RACHAELB@BU.EDU

**Role in Study (ie. Study Coordinator, Statistician, Study Nurse, MD, etc):**

A project specific Conflict of Interest Disclosure Form might be required for this person, to be submitted to the appropriate office.

**Did this person identify a conflict of interest in this form?**

○ Yes ◉ No

**BMC, Boston Public Health Commission (BPHC) or a Boston Healthnet Community Health Center (CHC)?**

◉ Yes ○ No

**For non-BUMC (non-BU/non-BMC) researchers ONLY, please indicate:**

**1. His/Her role in this study**
**2. Who will supervise his/her conduct in this study**
**3. Degrees achieved**
**4. Licenses held (e.g. MD, RN, RPh, LICSW, DMD/DDS, DO, other)**
**5. Professional liability insurance coverage (type and coverage limits)**
**6. University and/or hospital affiliations relevant to this study**
**7. Whether this researcher is considered "engaged" at his/her institution according to the OHRP Guidance.**

## 6.0 Conflict of Interest

### 6.1 Conflict of Interest Disclosure

By approving this protocol, as Principal Investigator, I am confirming that the **appropriate individuals** have filed a BU **Project Specific Disclosure (PSD)** with the **appropriate office**. I understand that this is a continuing obligation as **new** individuals join my research team in the future.

☑ Agree

**Of the BU PSDs submitted, have any *significant financial interests* been disclosed?**

○ Yes ◉ No

**If yes, please specify who has disclosed a COI.**

### 6.2 Conflict of Interest Disclosure imported from INSPIR I

This question is read-only. It has been replaced by the statement listed above.

BU and BMC policy requires that all internal investigators, and some external investigators, complete a project specific Conflict of Interest Disclosure Form and submit it to the appropriate office (click on the Help icon for instructions). Do not submit these forms to the IRB. Do not attach these forms to this protocol.

Have all investigators and staff in this study submitted COI Forms?

◉ Yes ○ No

# 7.0 Funding Source

### 7.1 Funding Source

What is the source of your research funding. If you have multiple sources of funding (including sub-awards), check all that apply.

- [ ] Unfunded Student Research
- [x] Dept/Internally Funded
- [ ] Government
- [ ] Industry
- [ ] Foundation/Other

### 7.2 Funding Details

For instructions on how to complete this section, click on the Help icon.

No Sponsors have been associated.

### 7.3 Grants Office

In the check boxes below, please indicate which grants office is handling your award/ sub-award.

- [ ] BU Office of Sponsored Programs (OSP-med)
- [ ] BMC Grants Administration (OGA)
- [ ] Charles River Campus Office of Sponsored Programs (OSP-CRC)
- [x] Other

BU-Bridge CTSI

**Funding Notifications:**

- [ ] I have received a Notification of Award (NoA)
- [ ] I have received a Just In Time notice (JIT)
- [ ] I have received a fundable score for this study.

### 7.4 Study Attachments

Click on the Help icon for information on what you're required to attach in this section.

No electronic document has been associated.

### 7.5 Funding Source Info from INSPIR I

This table is read-only. It will only be populated if this study was migrated from INSPIR I. If there are entries in this table, please use them to enter the funding information into the new Funding Source table above.

| Funding Type | Sponsor Name | Award # | PI of Award | Industry Protocol Number |
|---|---|---|---|---|
| Dept/Internally Funded | BU Bridge Pilot Grant | UL1RR025771 | | |
| Government | | | | |
| Industry | | | | |
| Foundation/Other | | | | |

**Industry Protocol Number:**

BU Source # or Record #:

BMC AU # or Record #:

ARRA Award:

☐ Please check here if this protocol will be funded through an ARRA award

## 8.0 Study Summary

**8.1 Provide a brief summary of the project in lay terms (in 300 words or less).**

Vitamin D deficiency is now recognized as one of the most common vitamin deficiencies in adults in the United States. Vitamin D deficiency has been connected to many chronic health diseases. The goal of this innovative research is to identify how vitamin D is able to have such wide ranging health benefits. This study will determine which genes are turned on and turned off in adults who receive 2000 IU vitamin D3 per day compared to 400 IU vitamin D3 per day. Results should provide important new insights about the health benefits of vitamin D for adults.

**8.2 Please skip this question for new studies - The following data was migrated from INSPIR I (if any). Eventually, the box below will go away. So please remove your answer from the box below and place it in the text editor (green button) above in section 8.1 by cutting and pasting it. The box below should be left blank.**

## 9.0 Study Site Information

**9.1 Select one:**

- ○ **Single site research** conducted by BUMC investigator(s) (skip question #2 below)
- ○ **Multi-site research project** - BUMC is a research site but is NOT the main study site (Skip question #2 below)
- ○ **Multi-site research** - BUMC is the main research site and/or BUMC investigator is the overall PI of the entire study or the FDA sponsor (must complete #2 below)

**9.2 Provide details of all other research sites involved in this study.**

| Institution & PI Information | IRB approval for site |
|---|---|

No records have been added

**9.3 Does this study involve Community Based Participatory Research?**

○ Yes ○ No

**9.4 Indicate below if any recruitment, consenting, and/or study interventions/procedures/data collection will take place in any of the following places (check all that apply)**

- [ ] Boston Healthnet Community Health Centers (click on ? icon for listing)
- [ ] MD offices or clinics (not part of BUMC campus)
- [ ] Subjects' places of residence including nursing homes, assisted living facilities, etc.
- [ ] Community centers or other 'community' locations (homeless shelters, daycare, etc.)
- [ ] International sites
- [ ] Veterans Administration (VA)

**9.5 Study Attachments**

Here you can attach any study sites related documents.
Attach IRB approval letters from other institutions (If you answered question #2).

No electronic document has been associated.

## 10.0 Navigation Menu

**10.1 Emergency Use**

**Is this application for an FDA approved EMERGENCY USE of an Investigational Drug or Device?**

○ Yes ◉ No

**10.2 Individual Patient IND or Humanitarian Use Device**

**Is this application for an FDA approved Individual patient (single use) IND or Humanitarian Use Device?**

○ Yes ◉ No

**10.3 Review Path Determination**

- ○ This project is exempt from IRB review because according to the regulatory definitions it is Not Human Subject Research (NHSR). Examples are Quality Assurance, Quality Improvement projects, or studies involving obtaining data/tissue.
- ○ Exempt - BUMC is Institution B
- ○ This project is exempt from BUMC IRB review because according to the Engagement of Institutions in Research guidance by OHRP neither BUMC (Boston University, Boston Medical Center) nor affiliated institutions/organizations for which the BUMC IRB has oversight responsibilities is ''engaged'' in human subjects research.
- ○ This study is exempt because all research activities fit into one or more of the Federal Exempt categories.
- ◉ This protocol cannot be exempt from IRB review.

**10.4 IRB Authorization Agreement (IAA) - BUMC is Institution A**

**Does this study have or require an IRB Authorization Agreement (IAA) where investigators <u>from another institution</u> will rely on BUMC IRB review ? *****

○ Yes ◉ No

**If this study has or will require an IRB Authorization Agreement (IAA) where BUMC investigators will rely on IRB review by another institution, do not check YES here, but instead, go to Exempt-BUMC is Institution B and check yes there.

***If the study is Exempt, then there should not be an IAA.

**10.5 International Research**

**Are any BU/BMC investigators involved in any way in any research activities at any non-US (international) sites, including oversight of international research activities?**

○ Yes ◉ No

**10.6 HIPAA Compliance**

**Is the PI a member of the covered entity and the study involves the collection of protected health information (PHI)? Is any investigator or member of the study staff, whether a member of the covered entity or not, using (i.e. accessing, recording) and/or disclosing PHI as part of this research?** If your answer to either question is YES then select Yes below.

◉ Yes - This study is subject to the HIPAA Privacy Rule

○ No - This study is HIPAA Exempt

**10.7 Genetics**

**Does this research involve genetic testing, gene therapy, or collection of genetic information?**

◉ Yes ○ No

**10.8 Biological Samples Collection**

**Does this study involve collecting, banking, and/or distributing biological samples?**

◉ Yes ○ No

**10.9 Drugs/Biological Agents**

**Does this study involve administering drugs or biological agents?**

◉ Yes ○ No

**10.10 Device**

**Does this study involve testing or use of a medical device?**

◉ Yes ○ No

**10.11 Repositories**

**Will you be collecting data or samples that will be placed into a repository, or will you be establishing a repository (either as a new protocol or to be added to an existing protocol)?** *(Do not check yes if this protocol involves ONLY obtaining samples FROM a repository to conduct this research)*

○ Yes ◉ No

**10.12 Study Finder**

**Would you like your study to be listed on a medical campus website that is available for public view (for purposes of recruitment, collaboration and publicity, etc.)?**

○ Yes ◉ No

## 11.0 Purpose

**11.1 Background/Rationale/Purpose**

Provide background information, study rationale, and purpose / study objective(s) and/or hypotheses for this study.

Vitamin D, commonly known as the sunshine vitamin, is produced in the skin

from sun exposure as well as from dietary sources. However, very few foods naturally contain vitamin D and the amount of vitamin D in fortified foods typically, 100 IU per serving, has been totally inadequate in satisfying adults vitamin D requirement, which is now been estimated to be at least 2,000 IU of vitamin D a day. As a result, vitamin D deficiency is rapidly being recognized world-wide as the most common vitamin deficiency. Upwards of 50-100% of children and adults have been reported as being vitamin D deficient depending on ethnicity, latitude and skin pigmentation. We reported in women at the time of delivery that 76% of mothers and 81% of newborns were vitamin D deficient despite the fact that the mother was taking a prenatal vitamin containing 400 IU vitamin D and drinking two glasses of milk a day. We also reported 30-80% vitamin D deficiency rates in white and black children, healthy young, middle aged and older adults. There have been numerous epidemiologic and clinical observations relating vitamin D deficiency to many chronic diseases and there are many isolated but no comprehensive studies evaluating various genes that are either suppressed or enhanced by 1,25-dihydroxyvitamin D [1,25(OH)2D]. It has been estimated that upwards of 2000 genes are directly or indirectly influence by 1,25(OH)2 D. To date, however, there have not been any genomic signatures identified in humans in response to correction of vitamin D deficiency. The goal of this pilot study is to determine whether or not vitamin D3 supplementation will affect biomarkers for calcium and bone metabolism, and how they alter gene expression biomarkers, especially genes related to the non-skeletal actions of vitamin D.

**11.2 Background/Rationale/Purpose - The following data was migrated from INSPIR I (if any). Eventually, the box below will go away. So please remove your answer (if any) from the box below and place it in the above text editor (green button) by cutting and pasting it. The box below should be left blank.**

## 12.0 Subjects

**12.1 Inclusion Criteria**

**Specify your inclusion criteria for each cohort.**

No Inclusion criteria has been associated.

The following data was migrated from INSPIR I (if any). Eventually, the box below will go away. So please remove your answer from the box below and place it in the above text editor (green button) by cutting and pasting it. The box below should be left blank.

Male and female adults of all races ages 18 years and older.

**12.2 Exclusion Criteria**

**Specify your exclusion criteria for each cohort.**

No Exclusion criteria has been associated.

The following data was migrated from INSPIR I (if any). Eventually, the box below will go away. So please remove your answer (if any) from the box below and place it in the above text editor (green button) by cutting and pasting it. The box below should be left blank.

1. Pregnant and lactating women.

2. Current or recent history of hepatic or renal disease

3. History of taking a daily supplement that contains more than 400 IU vitamin D2 or vitamin D3 within the past month or taking a pharmacologic amount of vitamin D2 or one of the active vitamin D analogs including Zemplar (Paricalcitrol), Dovonex (calcipotriol), Hectoral (vitamin D pro hormone)

4. Subjects who are taking antiseizure medications or glucocorticoids.

5. Exposure to a tanning bed or tanning on a beach for more than eight hours within the past month.

6. Known history of elevated calcium. (> 10.5 mg% (mg/dl))

7. History of intestinal malabsorption (i.e. Cystic Fibrosis, Fat Malabsorption Syndrome, Crohn's Disease)

8. Unwilling to consent to this trial

**12.3 Subjects (Please choose the appropriate categories for your subjects.)**

**Gender**
Both

**Age**

- [ ] Adolescent (15-17 years)
- [x] Adult (18-64 yrs)
- [ ] Child (7-15 years)
- [ ] Child < 7 years
- [ ] Fetus
- [x] Geriatric (65+ yrs)
- [ ] Other/unknown (specify in the box below)

**Race/Ethnicity:**

- [x] All Ethnic Groups
- [ ] American Indian or Alaskan Native
- [ ] Asian or Pacific Islander
- [ ] Black (Not of Hispanic Origin)
- [ ] Hispanic
- [ ] Mixed Race or Ethnicity
- [ ] White (Not of Hispanic Origin)
- [ ] Other or Not Available (specify in the box below)

**Languages: Remember that informed consent forms and all other written documents must be given in a language understandable to the subject. List all languages in which you are planning to obtain informed consent. Once the English version of the consent form is approved in INSPIR, please submit an Amendment with applicable translated consent & attestation forms prior to use.**

**Languages**

**Which if any of the following vulnerable populations will be recruited as subjects?**

- [x] BU Undergraduate Students
- [x] BUMC Students
- [ ] Children
- [ ] Children who are wards of the State
- [ ] Cognitively Impaired
- [ ] Emergency Department Patients
- [ ] BU/BMC Employees or lab personnel
- [ ] Homeless
- [ ] Individuals with Psychiatric Disorders
- [ ] Minors Independently making their own healthcare decisions
- [ ] Pregnant minors
- [ ] Pregnant Women
- [ ] Prisoners
- [ ] Terminally ill patients
- [ ] Wards of the State - Adults
- [ ] Women of Child bearing Potential

**12.4 Vulnerable populations require special protections. How will you obtain informed consent, protect subject confidentiality, and prevent undue coercion?**

There will not be direct contact from investigators. Recruitment will solely be based on response to flyers.

The following data was migrated from INSPIR I (if any). Eventually, the box below will go away. So please remove your answer (if any) from the box below and place it in the above text editor (green button) by cutting and pasting it. The box below should be left blank.

## 13.0 Design/Procedure

**13.1 Design**

**This study is:**

- ( ) Investigator initiated
- ( ) Sponsor initiated

**This study is:**

- ( ) Social /behavioral/educational research only (no biomedical interventions)
- ( ) Involves biomedical interventions and /or FDA regulated products (biomedical only)
- ( ) Combines biomedical and social behavioral aspects
- ( ) Chart/record/data base review only
- ( ) Repository only

**Data/ samples collected for this study involve:**

- ( ) Retrospective data/samples only
- ( ) Prospective data/samples only
- ( ) Retrospective and prospective data/samples

**13.2 Design - Read only. Migrated form INSPIR I.**

Categorize your research:
Specimen in vitro
Other:

**13.3 Discuss the research design including but not limited to such issues as: probability of group assignment, potential for subject to be randomized to placebo group, who is responsible for the randomization at local site, use of control subjects, etc.**

This is a randomized, controlled, double-blinded, investigator-initiated, single center pilot trial. We will recruit a total of 40 adult subjects for this study. Eligible participants that meet the inclusion/exclusion criteria will be enrolled in the study. We will recruit participants through flyers which will be posted around BUMC campus. The main objective in this pilot study is to determine whether or not enhanced vitamin D3 supplementation will improve biomarkers for calcium and bone metabolism, and favorably alter gene expression biomarkers, especially genes related to vitamin D non-skeletal actions. The 40 subjects will be randomly assigned to 1 of 2 groups (Group I and Group II) using a computer-generated randomization scheme. Group I: 20 subjects, who will receive 400 IU/d of vitamin D3 over a 2 month period Group II: 20 subjects, who will receive 2000 IU/d of vitamin D3 over a 2 month period Blood will be collected at the beginning of the study, before subjects are given vitamin D supplementation. Blood will also be collected at the end of the 2 month study.

The following data was migrated from INSPIR I (if any). Eventually, the box below will go away. So please remove your answer (if any) from the box below and place it in the above text editor (green button) by cutting and pasting it. The box below should be left blank.

**13.4 Procedure**

**Describe in detail the experimental design, including all materials and all procedures to be performed in sequential order as they will be performed. Clarify which procedures/test articles are investigational and which are part of standard clinical care. This description may include:**

1. methods
2. specific information concerning experimental interventions, such as dose and frequency of drug (and placebo) administration, or deception/debriefing process for social behavioral studies
3. number, frequency and duration of subject contacts (visits, telephone calls, mail outs, emails)
4. entire duration of participation for a single subject
5. any additional requirements of the subject (post treatment follow-up, diary cards, questionnaires, etc.)

**For multiple sites, indicate which of the procedures will be done at any other sites other than BUMC (see Study Site Information). Attach, in the Study Attachments section, copies of any surveys, questionnaires, and other data collection instruments.**

Before any study procedures are initiated for any subject in this study, an Institutional Review Board approved written informed consent form will be properly executed and documented. We will recruit 40 adult subjects. The 40 subjects will be randomly assigned to 1 of 2 groups (Group I and Group II) using a computer-generated randomization scheme. Group I: 20 subjects, who will receive 400 IU/d of vitamin D3 for a period of 2 months Group II: 20 subjects, who will receive 2000 IU/d of vitamin D3 for a period of 2 months The subjects will receive a 2 month supply of vitamin D3 capsules at their first visit to the GCRU. The vitamin D3 capsules will be provided by the BMC IDS pharmacy, under the direction of Dr. Hyeseon Hong. Blood will be collected at the beginning of the trial and at the end of the two months. We will ask the

subject to bring in the vitamin D3 bottles to count the number of remaining capsules, which will help with evaluating compliance and total amount of vitamin D3 taken by the subject. We will do a broad-gene expression screening of the subjects' buffy coats before and after treatment. The buffy coat is the layer of white blood cells between the sera and red blood cells. We will investigate whether genes associated with vitamin D action are being influenced by the increased intake of vitamin D and to uncover genes that may alter their expression that are indirectly influenced by enhanced vitamin D intake. Subjects will be asked to make 2 visits to the GCRU. Visit 1: During their first study visit at the GCRU we will collect: demographic data, body weight, height, BMI, past vitamin D use, urine pregnancy test (females only), current medication useage and/or anticipated medication usage during the study period. We will also collect 10 mL of blood which will be used to determine serum 25(OH)D, PTH, C-telopeptide of type I collagen (CTX), osteocalcin, calcium, phosphorus, and alkaline phosphatase. Another 10 mL of blood will be collected to obtain a buffy coat. The visit should take 45 minutes to complete. Visit 2: Visit 2 will occur 2 months after visit 1. Subjects will return with the vitamin D3 bottles so we can count how many capsules remain and determine compliance. We will collect body weight, height, BMI and record any adverse events. Subjects will be asked how many hours a week they spent outside and how often sunscreen was used. Data such as demographic data, body weight, height, BMI, past vitamin D use, current medication usage and sun exposure will be collected on data collection forms (refer to Section S). We will also collect 10 mL of blood which will be used to determine serum 25(OH)D, PTH, CTX, osteocalcin, calcium, phosphorus, and alkaline phosphatase. Another 10 mL of blood will be collected to obtain a buffy coat. The visit should take 30 minutes to complete. Sera collected for evaluation at Quest Diagnostics and the BUMC CORE lab will be coded with an identification number, and then will be stripped of identifiers. The information obtained for this study will be entered into an Excel spreadsheet which will be located in an electronic study folder on the desktop PC of one of the investigators. The sera will be stored in the respective labs located on the grounds of BUMC for up to five years. At that time the sera at BUMC will be discarded. To minimize sun exposure as a confounding factor, we will conduct the study in the winter months. The study will take no more than 2 years to recruit, enroll, analyze data and prepare the manuscript for publication. Due to the limited enrollment window of wintertime, we conservatively anticipate 2 winters to recruit and enroll 40 subjects. We also anticipate time to analyze the data and write the manuscript.

The following data was migrated from INSPIR I (if any). Eventually, the box below will go away. So please remove your answer (if any) from the box below and place it in the above text editor (green button) by cutting and pasting it. The box below should be left blank.

**Outcomes (Indicate anticipated primary and any secondary outcomes and how they will be measured):**

**Estimated Duration of Enrollment (Indicate how long will it take to recruit the required sample size):**

**Estimated Duration of Entire Study (Indicate estimated duration from initial IRB approval through data analysis to close of study):**

### 13.5 Study Attachments

*You must attach to this application all surveys, interviews, questionnaires, focus group outlines, etc. that will be used in this study. The IRB must review these materials as part of it review. If these items are included as part of the grant application they do not have to be submitted again. Failure to provide this information could result in a delay in IRB review. If some of the materials are not finalized- submit the DRAFT versions. The final versions will need to be approved by the IRB via an amendment PRIOR to use.*

No electronic document has been associated.

## 14.0 Sample Size/Specimens/Data Analysis

### 14.1 Sample Size (Click on the Help icon for instructions)

How many subjects (or records, or specimens, or charts) will be enrolled in this study?

| Subjects under BU/BMC PI (click on the Help icon for instructions) | For multi-center studies only - Total worldwide subjects, including subjects under BU/BMC PI |
|---|---|
| 40 | 40 |

**Subjects under BU/BMC PI Sample Size Calculations (Table's grand total should equal to the Subjects under BU/BMC PI sample size):**
If this protocol involves more than one cohort or study phase please specify anticipated sample size for each cohort /study phase.

| Cohort (study group) | Consent and/or fully participate in study | Expected dropouts, withdrawals, and terminations | Screened and not enrolled - for studies where subjects were placed `at risk` during screening (e.g., blood draws, collection of identifiable information) | Total for this cohort |
|---|---|---|---|---|

No records have been added

### 14.2 Sample Size Justification

Indicate why you chose the sample size proposed. Provide your sample size calculations. If this is a pilot study, this justification does not necessarily require a formal sample size calculation, but should provide a rationale for choosing the sample size proposed (e.g. to estimate a mean to a certain accuracy, to determine if the response rate is above a certain percentage, etc.) Note: Once the IRB approves a certain study sample size then you may not enroll beyond that sample size without first obtaining approval from the IRB. **** In determining your sample size be sure to allow for screen failures and study drop-outs. Explain how many evaluable subjects you will need to end up with to answer your study question and how many subjects you will need to enroll and consent to achieve this number. The IRB counts study subjects starting when they are screened/consented.

The 40 subjects we will enroll are part of a pilot study. We conservatively estimate that 10% of the subjects will drop out or be lost to follow-up, leaving us with data from 36 subjects to analzye. We believe it will take at least 18 subjects per group to generate statistically significant increases in serum 25 (OH)D levels. There have been some in vitro and animal studies that have looked at what genes are influenced by 1,25(OH)D2D but there have not been any human studies. We have no idea what genes will be affected in humans. Therefore, 20 subjects per group with a 10% drop-out rate should yield enough data to warrant further investigation. Prior studies that we have conducted,

have shown that an n of 20 per group will yield statistical significance for 25 (OH)D levels which are the only thing we can really analyze. Gene expression in humans consuming vitamin D has not been done, so we do not know which genes to look for or analyze but we are hoping this study will give us an idea of what genes are influenced by vitamin D so we can use the results from this study and apply it to further trials. Heaney RP et al, (Heaney RP Davies KM, Chen TC, Holick MF, Barger-Lux MJ 2003 Human serum 25-hydroxycholecalciferol response to extended oral dosing with cholecalciferol. Am J Clin Nutr 77:204¿210) showed that serum 25(OH)D levels increased by 1 ng/ml for every 100 IU vitaminD3. Accordingly, assuming this mean change of 20 ng/ml and 4 ng/ml over 8 weeks following doses of 2000 IU of vitamin D3 and 400 IU of vitamin D3 respectively, samples of 20 subjects per group provide 90% power of showing a greater increase in 25(OH)D in high dose (2000 IU) vs low dose (400 IU) (via the two-sample t-test on changing scores, testing at the two-tailed p<0.05 level).

The following data was migrated from INSPIR I (if any). Eventually, the box below will go away. So please remove your answer (if any) from the box below and place it in the above text editor (green button) by cutting and pasting it. The box below should be left blank.

**14.3 Data Analysis**

Provide a description of your plan for data analysis. State the types of comparisons you plan (e.g. comparison of means, comparison of proportions, regressions, analysis of variance). Which is the PRIMARY comparison/analysis? How will the analyses proposed relate to the primary purposes of your study? If you are doing qualitative research please state how comparisons will be made.

Gene expression in humans consuming vitamin D has not been done, so we do not know which genes to look for or analyze but we are hoping this study will give us an idea of what genes are influenced by vitamin D so we can use the results from this study and apply it to further trials. One of our objectives is to look at mean changes of 25(OH)D between the group receiving 2000 IU vitamin D3 and 400 IU vitamin D3. Changes in circulating levels of 25(OH)D with vitamin D-enhanced supplementation compared to the standard vitamin D dose will be characterized by two-tailed t-test to evaluate differences in the effect of vitamin D3-enhanced supplementation between treatment groups. Comparison within each group from baseline to the end of the study will be run using an independent t-test for continuous outcomes.

The following data was migrated from INSPIR I (if any). Eventually, the box below will go away. So please remove your answer (if any) from the box below and place it in the above text editor (green button) by cutting and pasting it. The box below should be left blank.

## 15.0 Potential Risk/Discomforts

**15.1 Lists the possibilities for risks of harm or discomfort to subjects as a result of their participation in the research.**

Risks to the subject due to study participation: The overall risk to the subject

due to participation in this study is minimal, as discussed below. Risk of phlebotomy: The risk of phlebotomy includes bruising, pain, and infection at the site of venipuncture. Blood drawn for involvement in this study will be drawn by a research member with experience in phlebotomy, thus minimizing the risk. Risk of breach in confidentiality: Multiple steps will be taken to ensure that this risk is kept to a minimum as outlined above. The data will be maintained by Dr. Michael F. Holick. There will be no paper hardcopy of the data. All data will be maintained on a personal computer in Room 1012 on the 10th floor of 85 E Newton St. This computer is password protected for further safety and the room is locked when study personnel are not present. Sequential numbers will be used to code for the privacy and unique identification of the research subjects. All information collected will be stripped of identifiers, and maintained in password protected files. Risks of overdosing of Vitamin D: Early signs and symptoms of overdose: Hypercalcemia, constipation, increased thirst, increase in frequency of urination, especially at night, or in the amount of urine. Late signs and symptoms of overdose: Hypercalcemia, constipation, calcium deposits in tissues outside of the bone, cloudy urine and drowsiness. Late symptoms of severe overdose: High blood pressure, irregular heartbeat, renal failure. There is essentially no risk for vitamin D toxicity from this dose of vitamin D. There have been no reports of toxicity for adults who take a single dose of 50,000 IU vitamin D or 10,000 IU vitamin D per day for up to 5 months. Risks associated with vitamin D supplementation: Vitamin D supplementation is associated with a very low frequency risk for significant adverse effects. In a study of healthy men given vitamin D3 orally up to 50,000 IU/day for 8 weeks, no significant adverse events were noted. At this dose, there have been no reported cases of untoward consequences. Therefore, a data safety and monitoring board is not required. The principal investigator, Dr. Michael Holick, will conduct oversight of the study, in an ongoing fashion. Data that will be reviewed for safety monitoring will include: adverse event/significant adverse effect reports per BUMC policy, subject's medical history, and laboratory data (specifically as listed in the protocol). There are not any drug interactions that subjects should be aware of.

**15.2 Provide a description of how risks will be minimized.**

**15.3 Description of Risks or Discomforts - The following data was migrated from INSPIR I (if any). Eventually, the box below will go away. So please remove your answer (if any) from the box below and place it in the appropiete sections listed above by cutting and pasting it. The box below should be left blank.**

In order to approve a study the IRB must be able to determine that *"Risks to subjects are minimized by using procedures which are consistent with sound research design and which do not unnecessarily expose subjects to risk."*

**List the possibilities for risk or harm to the subjects as a result of their participation in the research.** Be sure to include physical harms, discomforts, hazards, inconveniences, or the potential for legal or social harms (i.e. loss of confidentiality). Whenever possible, include for each:

1. Probability of occurrence
2. Magnitude

3. Duration

**For each harm listed, indicate measures that will be taken to prevent or minimize the effects of all of the potential the hazards, discomforts or risks.**

---

# 16.0 Data & Safety Monitoring

**16.1 Data and Safety Monitoring Plan (DSMP)**

CLICK ON THE HELP ICON (?) FOR MORE INFORMATION ABOUT DSMPS

**16.2 Adverse events (AEs),serious adverse events (SAEs), Unanticipated Problems (UPs). (Check all that apply)**

- ☐ AEs, SAEs, are defined in an attached detailed protocol.
- ☐ This is not a drug/device study or an intervention study. Only AEs/SAEs and UPs that are related or possibly related to the research will be collected and reported.
- ☐ This is a survey/interview/observational study. The only risks are related to confidentiality. No AEs/SAEs will be reported unless they meet the definition of an UP. Security /confidentiality breaches will be reported to the IRB as UPs.
- ☐ A DSMP has been created using the BUMC DSMP template and attached in the Study Attachments section below.
- ☐ Other definitions will be used for AEs/SAEs, and UPs. Describe below.
- ☐ We will NOT follow the BUMC policy for reporting AEs/SAEs and UPs. Describe alternate plan below.

*Unless specified the expectation is that BUMC policy will be followed for reporting AEs, SAEs, and UPs. Click here for link to BUMC policy

**16.3 Frequency of monitoring. How often will the data be monitored by the entity/entities selected in question above? Provide additional details in the text box below.**

- ○ DSMB/DMC/Independent Monitor will provide written reports annually
- ○ DSMB/DMC/Independent Monitor will provide written reports every 6 months
- ○ Other details about monitoring activities including by CRO & sponsor (describe below)

---

**16.4 Stopping rules: for individual subjects and for the study as a whole. Not all studies require stopping rules. Describe any stopping rules in the box below.**

**16.5 Study Attachments**

Here you can attach any Data and Safety Monitering Plan documents including BUMC DSMP template, DSMB charter, and any other related documents.

No electronic document has been associated.

**16.6 Read-only. This question was migrated from INSPIR I.**

**Outside/ Independent Monitors**

**For some studies, for example, those that are moderate to high risk, the IRB may require data/safety review by an outside monitor.** ***( check all that apply)***

This study is a minimal risk study and no independent monitoring is required.

○ Yes ○ No

This study will have an independent Data and Safety Monitoring Board. If yes, attach the DSMB charter.

○ Yes ◉ No

This study will have an independent Data Monitor. If yes, insert information about the monitor in the box below.

○ Yes ○ No

This study will be monitored by a clinical research monitoring organization CRO. If yes, specify details in the box below.

○ Yes ○ No

Independent Data Monitor and/or CRO Details:

## 17.0 Potential Benefits

**17.1 Describe potential benefit(s) to be gained by the individual subject as a result of participating in the research. (Payments to subjects should not be included in this section.)**

By taking vitamin D3, subjects will increase their serum 25(OH)D levels for the two months they are in the study.

The following data was migrated from INSPIR I (if any). Eventually, the box below will go away. So please remove your answer (if any) from the box below and place it in the above text editor (green button) by cutting and pasting it. The box below should be left blank.

## 18.0 Potential Benefits - Cont.

**18.1 Describe potential benefit(s) to society and scientific/medical knowledge in the research.**

There is a great need to know how much vitamin D adults need for maximum health. There is very scarce information about a dose relationship of vitamin D, either on calcium metabolism and skeletal health, or on the nonskeletal benefits of vitamin D, including reducing risk of autoimmune diseases, respiratory tract infections, wheezing illnesses, asthma, as well as type II diabetes and hypertension. In this pilot study, we will be able to answer a fundamental question about vitamin D nutrition. What will be the effect of increasing the vitamin D3 intake by 400%, from 400 IU to 2,000 IU of vitamin D3 a day for two months on circulating levels of 25(OH)D and parameters of calcium and bone metabolism. In addition, we will do a broad-gene expression screening of their buffy coats before and after treatment investigating whether genes associated with vitamin D action are being influenced by the increased intake of vitamin D and to uncover genes that may alter their expression that are indirectly influenced by enhanced vitamin D intake.

The following data was migrated from INSPIR I (if any). Eventually, the box below will go away. So please remove your answer (if any) from the box below and place it in the above text editor (green button) by cutting and pasting it. The box below should be left blank.

**18.2 Risk to Benefit Ratio**

**Describe how risks to subjects are reasonable in relation to anticipated benefits:**

In a study of healthy men given vitamin D3 orally up to 50,000 IU/day for 8 weeks, no significant adverse events were noted. In this study, control subjects will receive 400 IU of vitamin D3 per day for a total of 2 months. 400 IU/d of vitamin D is twice the adequate intake recommended for adults up to 50 years of age and therefore it is highly unlikely that cause any adverse events. It was recently documented that people over the age of 12 can receive 2,000 IU of vitamin D3 a day without any concern for toxicity. The benefits include: discovering whether genes associated with vitamin D action are being influenced by the increased intake of vitamin D, uncovering genes that may alter their expression that are indirectly influenced by increased vitamin D intake.

The following data was migrated from INSPIR I (if any). Eventually, the box below will go away. So please remove your answer (if any) from the box below and place it in the above text editor (green button) by cutting and pasting it. The box below should be left blank.

## 19.0 Recruitment Procedures/Materials

### 19.1 Recruitment Procedures

Who will recruit subjects for this study?

- [x] PI
- [x] PI's Staff
- [ ] Research subject (e.g., recruitment of family member into genetic studies)
- [ ] Third Party

Third Party Info:

**Describe in detail how the research population will be identified and your methods for contacting potential subjects. If this study is a chart review or medical record review, explain how you will identify potential records to be reviewed.**

We will place a flyer on the Boston University Medical Center campus to recruit study participants (see Section R and flyer attached to Section S). This will instruct interested parties to contact Rachael Biancuzzo for further details and to determine eligibility.

The following data was migrated from INSPIR I (if any). Eventually, the box below will go away. So please remove your answer (if any) from the box below and place it in the above text editor (green button) by cutting and pasting it. The box below should be left blank.

### 19.2 Recruitment Material

Add any recruitment material that will be used in the table below.  If a video, submit the tape.  If a website, provide the URL.

**Recruitment Materials**

**Recruitment Materials**

Attach in the Study Attachments section, copies of materials such as: posters, flyers, newspaper ads, script for telephone recruitment. If a video, submit the tape. If a website, provide the URL.

**Recruitment Material: BMC Health Connections Website**

Mode of Recruitment:

Flyer

Other Recruitment Material:

Exact Language of Recruitment Material:

ARE YOU VITAMIN D DEFICIENT?

We are looking for:

Normal volunteers without any history of gastrointestinal or malabsorption disorders.

Purpose:

The purpose of this study is to whether or not enhanced vitamin D3 supplementation will improve biomarkers for calcium and bone metabolism, and favorably alter gene expression biomarkers. Study participants must be 18 years of age or older.

Participation in the study involves 2 visits to the GCRU and 2 blood draws.

Compensation of $50 for completing the study will be provided. ($25 will be prorated for the first visit if the subject is unable to complete the entire study.) Parking vouchers available.

For more details, please contact: Rachael Biancuzzo 617.638.8860

**19.3 Screening**

Will there be any screening procedures done to determine subject eligibility in this study?

○ Yes ◉ No

**19.4 Study Attachments**

Here you can attach any study related documents including, but not limited to, recruitment material related documents.
Please attach copies of materials such as: posters, flyers, newspaper ads, script for telephone recruitment (if any).

No electronic document has been associated.

## 20.0 Consent Procedures

**20.1 Consent Procedures**

Will informed consent be obtained for this research?

○ No (skip the follow up question below)
○ Yes (answer the follow up question below)

If yes, describe in detail the informed consent process, i.e. who will obtain consent and where, how long will subjects have to consider participating, is consent required prior to eligibility screening. If children will be enrolled, describe the assent process.

Consent would be obtained by Dr Holick, Dr. Demetriou, Rachael Biancuzzo, Timothy Calvert, Lenore Dacuycuy, Cassandra Voltaire, Esther Kim, Joe Lytle or Hataikarn Nimitphong at the time of the subject's first visit to the GCRU. The subjects will have sufficient time to read the informed consent form, and to ask questions about the study during the initial screening visit. Furthermore, patients may withdraw from the study at any time, for any reason.

The following data was migrated from INSPIR I (if any). Eventually, the box below will go away. So please remove your answer (if any) from the box below and place it in the above text editor (green button) by cutting and pasting it. The box below should be left blank.

**20.2 Verbal Consent/Assent - Waiver of Documentation of the Informed Consent**

Will this research include an informed consent process, but require a Waiver of Requirement for Documentation of Consent?

○ Yes ◉ No

If yes, please explain in the text box below how your study meets one of the two criteria in 45 CFR 46.117(c) (see ? for criteria):

The following data was migrated from INSPIR I (if any). Eventually, the box below will go away. So please remove your answer (if any) from the box below and place it in the above text editor (green button) by cutting and pasting it. The box below should be left blank.

**20.3 Waiver of Informed Consent Process**

Will this research require a Waiver of Informed Consent?

○ Yes ◉ No

If you are requesting that the IRB approve a Waiver of Consent (you will not obtain informed consent) indicate this in the text box below. Explain specifically why you will not obtain consent. Provide as much information as possible to allow the IRB to make a determination based on the required criteria 45 CFR 46.116(d)(1-4). (Click on ? for criteria)

The following data was migrated from INSPIR I (if any). Eventually, the box below will go away. So please remove your answer (if any) from the box below and place it in the above text editor (green button) by cutting and pasting it. The box below should be left blank.

**20.4 Assent (from Minors)**

Indicate in the text box below if you intend to obtain assent from minor subjects. As a rule the IRB requires verbal assent for minors 7-11 years of age and written assent from minors ages 12-17 Note: if verbal consent is approved by the IRB for the parents/adult subjects (see the Verbal Consent/Assent section above), then verbal assent may be allowed also for 12-17 year olds. ** Be sure to discuss any plans for obtaining consent/assent from pregnant minors.

**20.5 Consent by Substituted Judgment**

Indicate in the text box below if you intend to obtain consent from a legally authorized representative for cognitively impaired/decisionally impaired subjects. Be sure to include information about how you will ascertain whether or not subjects are capable of consenting themselves and how you will determine who may provide consent for them. ***Note : consent can only be obtained from someone other than the subject with specific IRB approval.

**20.6 Non-English Language Consent Forms:**

Will this study require one or more non-English language consent forms?

○ Yes ○ No

If you answered yes above, for each Non-English language you listed in the Subjects section, add the language to the table below and indicate which consent document you will use:

| Language | Translation |
|---|---|
| No records have been added | |

**Non-English Language Consent Attachments**

Attach here any documents and forms related to the non-English language consent process, click on the (?) icon for instructions and related forms. Attach here such documents as the Request For Use of Short Consent Form Process, the Short Consent Form in English, and the Short Consent Form in any of the available non-English languages.

No electronic document has been associated.

---

**The following two text boxes are read only. They are data migrated from INSPIR I (if any) to assist you in answereing the rest of this section above.**

Translation of the entire Consent Form

Short Form Consent Document

## 21.0 Confidentiality

**21.1 Confidentiality**

Will research data include elements which will allow the subjects to be identified?

◉ Yes ○ No

**Confidentiality of the Data**

State what steps will be taken to maintain confidentiality of data and privacy (or anonymity) of subjects. *Specify whether study data will be identified by specific subject identifiers (name, medical record numbers, etc.) or by study IDs that can be linked to subject identifiers via a master-code or key.*

The information obtained will be entered into the computer of one of the investigators. To ensure privacy, each patient will be assigned an identification

number, and then will be stripped of identifying information (i.e. name, date of birth, medical record number, etc.) A master list of patient names, medical record numbers and identification numbers will be maintained on a separate Excel spreadsheet. The PC will be password protected to further ensure privacy.

The following data was migrated from INSPIR I (if any). Eventually, the box below will go away. So please remove your answer (if any) from the box below and place it in the above text editor (green button) by cutting and pasting it. The box below should be left blank.

**Please check all that apply:**

- ☐ Study data will be anonymous. All data will be RECORDED as anonymous. There will be no way to link data to individual subjects, even temporarily AND subjects' identities cannot be reasonably ascertained via deductive disclosure.
- ☐ Study data will be coded. All study documents will be identified by a unique study ID. The unique study ID will be linked to subject identifiers via a mastercode or key. Access to the mastercode/key will be limited to the researchers. The mastercode/key that links study data to identifiers will be stored separately from the study data and protected (locked, separate flash drive, etc.)
- ☐ Study data will contain certain identifiers such as dates including dates of birth, medical record numbers, etc. Data will not contain social security numbers.
- ☐ Study data will contain high risk identifiers (e.g. social security numbers) or very sensitive information with subject identifiers such as HIV status, psych diagnosis, illegal drug use, etc.
- ☐ There is an alternate plan for how subjects will be identified in study documents. Please specify in text box below.

**Release of identifiable data.**
Indicate who will be PROVIDED with identifiable research data (including "coded" data). Be sure to include study sponsors, students, outside institutions, etc. (Note: in most instances NIH and other study sponsors are **not provided** identifiable study data but they **have access to study data** on-site for monitoring and auditing purposes. The IRB and the other institutional officials also have access to study data for audit and quality assurance purposes. These do not have to be listed below) Include any release of study data into registries or research databases.

| Who gets data | Type of data |
|---|---|

No records have been added

Read-only migrated from INSPIR I.

Besides the PI, study staff, and the IRB, no one else will have access to identifiable research data

**Storage and destruction of study data**
Where will research data be kept? How will such data be secured? How long will it be kept? How and when will it be destroyed?

- *Note: Federal regulations require that study data be maintained by the investigator for a minimum of three years following the COMPLETION of the study. FDA regulations may require that study data be retained for significantly longer.*

The information obtained will be entered into the computer of one of the investigators. To ensure privacy, each patient will be assigned an identification number, and then will be stripped of identifying information (i.e. name, date of birth, medical record number, etc.) A master list of subject names and identification numbers will be maintained on a separate Excel spreadsheet. The computer will be password protected to further ensure privacy. The information will be kept for 3 additional years post conclusion of the study, and will at that time be erased from the memory of the computer. No paper records will be maintained.

The following data was migrated from INSPIR I (if any). Eventually, the box below will go away. So please remove your answer (if any) from the box below and place it in the above text editor (green button) by cutting and pasting it. The box below should be left blank.

**Certificate of Confidentiality**
Will you obtain a Certificate of Confidentiality for this study?
*(Note: If a CoC will be obtained then CoC language is required in the consent form. See IRB website for more information about CoCs.)*

○ Yes ◉ No

## 21.2 Study Attachments

Here you can attach the Certificate of Confidentiality (CoC) and any Confidentiality related documents.

No electronic document has been associated.

# 22.0 HIPAA Compliance

### 22.1 HIPAA

**Indicate below all forms that apply to the research. All forms selected below, except for the HIPAA Authorization, need to be downloaded from the help icon, filled out, and attached to the protocol in the Study Attachments section:**

- [x] HIPAA Authorization (PI will include HIPAA Authorization language in the Consent Form)
- [ ] Waiver of Authorization Form
- [ ] De-identified Data Form
- [ ] Limited Data Set Form
- [ ] Preparatory to Research Form
- [ ] Decedent Research Form

### 22.2 Study Attachments

**All HIPAA forms selected above, except for the HIPAA Authorization, need to be attached to the protocol in this section.** Click on the Help (?) icon for a list of these forms, then click on the form's link to download and save a copy on your desktop. After completing the form, upload it in this section.

No electronic document has been associated.

# 23.0 Cost/Payment

**23.1 Cost**

What costs / potential costs will subjects incur (include travel, parking, medication, etc.)? How will the cost of research visits / procedures be covered? Will the subject (or the subject's insurance) be responsible for any research related costs? If yes, state specifically which items the subject (or the subject's insurance) will be responsible for and the cost of each.

No additional expenses due to involvement in this research study will be incurred by the research participant or the participant's insurance company.

**23.2 Payment / Course Credit**

**Payments**

If subjects will be paid (money, gift certificates, coupons, etc.) to participate in this research project, please note the total dollar amount (or dollar value amount) and distribution plan (one payment, pro-rated payment, paid upon completion, etc.) of the payment. Describe any other reimbursement that will be provided to subjects, (i.e. travel, parking, public transportation, etc.). Explain specifically how and when these reimbursements for expenses will be paid. Specify your plan for reimbursement if a subject withdraws from the study.

In order for it to be more likely to recruit sufficient numbers of volunteers, a financial incentive of $50 will be provided for completing the entire study. Each visit the subjects complete will be prorated at $25. Parking vouchers for volunteers will be available if they park in the BU garage during their study visit.

**Course Credit -** If student subjects will receive course credit for their participation in this study. Explain below.

## 24.0 Genetics

**24.1 How would you classify/describe the genetic research component of this research protocol?**

- ☐ Gene transfer or gene therapy (these studies require review by the BUMC Human Gene Therapy Committee)
- ☐ Pedigree study (to discover the pattern of inheritance of a disease and to catalog the range of symptoms)
- ☐ Positional cloning (to localize and identify specific genes)
- ☑ DNA Diagnostic study (to develop techniques for determining the presence of specific DNA mutations or polymorphisms)
- ☐ Association Studies (genotype-phenotype correlation) including GWAS (genome wide association studies)
- ☐ Genetic pharmacokinetic research
- ☐ Other (specify in text box below)

**24.2 CLIA-certification for genetic tests?**

- ○ All genetic testing where results will be used for clinical decision-making or reported to subjects and/or their clinicians will be done in a CLIA-certified lab
- ○ No genetic test results will be given to subjects or their clinicians
- ○ Other (specify below)

**24.3 What are the plans for the use of the samples collected for genetic testing? (Indicate all that apply and provide additional details in text box below)**

- ☐ Genetic samples will be/may be used to develop cell lines

- [ ] Genetic samples may have identifiers removed and sold commercially or given to commercial sponsors
- [ ] Genetic samples will be/may be saved in a repository for future genetic testing
- [ ] Genetic samples will be used for genetic testing ONLY for the purpose of this study and all left-over genetic samples will be destroyed

**24.4 Genetic Data**

What are the plans for use of the genetic information collected? (indicate all that apply and provide details in text box below)

- [ ] Genetic data will be used for this study ONLY to meet the goals of this study
- [ ] Use of genetic data will be limited to use by BUMC investigators for research related to this study and consistent with the consent form. (Those studies will require a separate IRB submission)
- [ ] Genetic data will be made available to investigators from other institutions or will be made publically available (specify below)
- [ ] Genetic information will be put into dbGaP or other central repository for use by other researchers. (specify below)
- [ ] Other (specify below)

**24.5 Sharing of Genetic Information**

If genetic information will be released to anyone, what are the plans for de-identifying the data? For all instances, specify by name who will be responsible for protection of the master-code and managing the release of the genetic data outside the institution. (provide details in text box below)

- ○ Data will retain subject identifiers (e.g. names, MR #, SS#, DOB, other dates)
- ○ Genetic information will ONLY be labeled with a study ID number. The BUMC PI will hold the master-code linking the study ID to the subjects' identifiers. The master-code will be stored separately from the subject identifiers and the master-code will NEVER be given to researchers outside the institution or to study sponsors
- ○ The data will be stripped of all identifiers that would allow subjects to be identified (will have no names, MR#, SS#, full dates or other identifiers that would allow for deductive disclosure)

**24.6 Secondary Use of Data**

If archived samples will be used for genetic testing, please explain in the text box below how, where, and why the original tissue was collected; were the samples obtained as part of clinical care or research; was the sample collection approved by an IRB; did the original consent cover secondary use; and were there any limitations on secondary use? Please attach a copy of the original ICF.

**24.7 Genetics Related Risks**

In genetic research, the primary risks, outside of gene therapy, are **psychological** and **socioeconomic**. Address the psychological and socioeconomic risks related to generating personal genetic information. Address whether or not genetic counseling service would be available for the participants.

The patient data will not available to the patient or a third party. The investigators will not be able to trace the patient to match the data.

The following data was migrated from INSPIR I (if any). Eventually, the box below will go away. So please remove your answer (if any) from the box below and place it in the above text editor (green button) by cutting and pasting it. The box below should be left blank.

*The consent form builder will contain template language to be added to consent documents for genetic research.

**24.8 Read-Only. This question was migrated from INSPIR I.**

Will subjects be provided with genetic results? Will subjects be offered any type of genetic education or counseling, and if so, who will provide the education or counseling and under what conditions will it be provided? If there is the possibility that a family's pedigree will be presented or published, please describe how you will protect family members' confidentiality.

No, the subjects will not be provided with the genetic results. They will not be offered any type of genetic education or counseling and a family pedigree will not be presented or published.

## 25.0 Biological Sample Collection

**25.1 Add New Samples**

### Sample Collection

**Sample**

**Select a sample type:**

Blood

Other:

What is the purpose of the sample collection?

We will collect the blood to determine serum levels of 25-hydroxyvitamin D [25(OH)D], PTH, calcium, alkaline phosphatase, phosphorus, osteocalcin and CTX. We will also use the blood to obtain a buffy coat to look at gene expression.

For blood draws, specify the amount drawn at each visit and across the course of the subject's entire participation time.

We will collect blood at the beginning and end of the two month trial.

We will collect a total of 40 mL of blood.

Visit 1: 10 mL will be used for determining serum levels of 25(OH)D, PTH, calcium, alkaline phosphatase, phosphorus, osteocalcin and CTX. 10 mL of blood will be collected and quickly processed to obtain the buffy coat.

Visit 2: 10 mL will be used for determining serum levels of 25(OH)D, PTH, calcium, alkaline phosphatase, phosphorus, osteocalcin and CTX. 10 mL of blood will be collected and quickly processed to obtain the buffy coat.

Is there the possibility that cell lines will be developed with this sample?
If you answer yes, then the cell line disclaimer language will appear on your consent form(s). Please review your consent form(s) in section Q before submitting.

○ Yes ◉ No

Sample will be obtained from:

- [x] Directly From Subject
- [ ] Pathology Department
- [ ] Clinical Labs
- [ ] Research Labs
- [ ] Other:

Will the sample be stripped of identifiers? An identifiable sample is any sample accompanied by codes

or data that could facilitate 1) re-contacting the subject or 2) gaining access to identifiable private information about the subject.

◉ Yes ○ No

**If sample will be released outside BU/BUMC:**

Will sample be released to anyone not listed as an investigator on the protocol? Will the information be identifiable, coded or de-identified? If coded, who will hold the code? How will it be secured? When/how will it be destroyed?

Coded samples will be sent to GCRU Core Lab in order to perform the vitamin D assay. The code will be held by Dr. Tai Chen in a separate password protected computer file. Blood will be sent to BMC to measure serum calcium and albumin. Leftover samples from the assays will be banked for 5 years for the purpose of repeating the assay with other methodologies such as liquid chromatography-mass spectroscopy (LC-MS/MS) if developed. LC-MS/MS is the contemporary gold standard assay for 25-hydroxyvitamin D analysis.

Will sample material be sold or transferred to any third parties? If so, describe the recipient. Will the information be de-identified? If so, describe how.

No third parties will be involved.

**If sample will be banked for future use:**

Where will the sample be banked and for how long? Will the subject be re-consented for future use?

Coded samples will be sent to GCRU Core Lab in order to perform the 25(OH)D assay. The code will be held by Dr. Tai Chen in a separate password protected computer file. Leftover samples from the assay performed at the GCRU Core Lab will be banked for 5 years for the purpose of repeating the assay with other methodologies such as LC-MS/MS if developed.

Does the banking institution have an IRB approved policy for the distribution of samples?

Samples will be sent to the GCRU Core Lab at Boston University Medical Campus (BUMC). Leftover samples from the assay performed at the Core Lab will be discarded after 5 years.

**If the entire sample will NOT be used during the course of this research study:**

Will the remaining sample be discarded? If not what will be done with the remaining sample after study completion and how long will the sample be kept?

The remaining sample at BUMC will be discarded after 5 years.

Will samples be made available to the research subject (or his/her medical doctor) for other testing?

○ Yes ◉ No

**If a subject withdraws from the study:**

Will the subject have the option to get the remaining portion of his/her sample back?

○ Yes ◉ No

Will the remaining sample be discarded? If not, will it be kept anonymously? What will happen to the sample if the subject revokes authorization?

The remaining samples will be discarded.

Will data obtained from the sample be deleted? What will happen to the data if the subject revokes authorization?

Any data collected before the patient withdraws from the study will not be deleted, as previously agreed. Should the patient revoke authorization, no further data will be obtained from patient or patient's samples.

Will study data or test results be recorded in the subject’s medical records?

○ Yes ◉ No

Will results of specific tests and/or results of the overall study be revealed to the research subject or his/her doctor?

Subjects will not be informed of their test results, unless they wish to know. The tests are run at the Core Lab at BUMC and at Quest Diagnostics Laboratory in Cambrigde, MA. Both the Core Lab and Quest are CLIA-certified. We will inform the subjects of their 25(OH)D, PTH, calcium, alkaline phosphatase, phosphorus, osteocalcin and CTX if they wish to know.

Please identify all third parties, including the subject's physician, to receive the test results.

No third parties will receive the test results unless per request of the patient.

## Sample Collection

**Sample**

**Select a sample type:**

Urine

Other:

What is the purpose of the sample collection?

To determine pregnancy status of women being screened for the study. Women who are pregnant will be excluded from the study.

For blood draws, specify the amount drawn at each visit and across the course of the subject's entire participation time.

N/A

Is there the possibility that cell lines will be developed with this sample?
If you answer yes, then the cell line disclaimer language will appear on your consent form(s). Please review your consent form(s) in section Q before submitting.

○ Yes ◉ No

Sample will be obtained from:

- [x] Directly From Subject
- [ ] Pathology Department
- [ ] Clinical Labs
- [ ] Research Labs
- [ ] Other:

Will the sample be stripped of identifiers? An identifiable sample is any sample accompanied by codes or data that could facilitate 1) re-contacting the subject or 2) gaining access to identifiable private information about the subject.

○ Yes ◉ No

**If sample will be released outside BU/BUMC:**

Will sample be released to anyone not listed as an investigator on the protocol? Will the information be identifiable, coded or de-identified? If coded, who will hold the code? How will it be secured? When/how will it be destroyed?

Sample will not be released outside of BU/BMC

Will sample material be sold or transferred to any third parties? If so, describe the recipient. Will the information be de-identified? If so, describe how.

No third parties will be involved.

**If sample will be banked for future use:**

Where will the sample be banked and for how long? Will the subject be re-consented for future use?

The sample will not be banked for future use.

Does the banking institution have an IRB approved policy for the distribution of samples?

N/A

**If the entire sample will NOT be used during the course of this research study:**

Will the remaining sample be discarded? If not what will be done with the remaining sample after study completion and how long will the sample be kept?

The remaining sample will be discarded after it is used to determine pregnancy status.

Will samples be made available to the research subject (or his/her medical doctor) for other testing?

○ Yes ◉ No

**If a subject withdraws from the study:**

Will the subject have the option to get the remaining portion of his/her sample back?

○ Yes ◉ No

Will the remaining sample be discarded? If not, will it be kept anonymously? What will happen to the sample if the subject revokes authorization?

Urine samples will be immediately discarded once pregnancy status is determined.

Will data obtained from the sample be deleted? What will happen to the data if the subject revokes authorization?

Any data collected before the patient withdraws from the study will not be deleted, as previously agreed. Should the patient revoke authorization, no further data will be obtained from patient or patient's samples.

Will study data or test results be recorded in the subject's medical records?

○ Yes ◉ No

Will results of specific tests and/or results of the overall study be revealed to the research subject or his/her doctor?

Results of the pregnancy test will be revealed to the research subject, with recommendation that she follows up with her PCP or OB/GYN if the pregnancy test is positive.

Please identify all third parties, including the subject's physician, to receive the test results.

No third parties will be involved.

## 26.0 Drug or Biological Agents

**26.1 Drugs or Biological Agents Information:**

Please list all drugs/biological agents administered in this study. If this study was migrated from INSPIR I, please re-list here all drugs/ biological agents that were listed in the read only questions shown above.

No Drug has been associated.

**26.2 Study Attachments**

Here you can attach any study related documents including, but not limited to, drugs/biological agents related documents.
*Please attach a copy of the investigator's brochure or the drug package insert for each drug listed above (if any).*

No electronic document has been associated.

**26.3 The next four questions and answers were migrated from INSPIR I and are read-only. They can be used to help you complete the questions listed above.**

For BUMC investigators, who is responsible for the storage and documentation of the drugs / biological agents used in this study?

- [x] Investigational Drug Service at BUMC
- [ ] The Investigator

IND Number:

**26.4 Name of the drug / biological agent, the name of the manufacturer and who is holding the IND.**

n/a

**26.5 Does this research involve a NEW use of an approved drug? If yes, insert the IND number in the IND box above and insert the drug/biological agent name, the name of the manufacturer, and who is holding the IND in the text box below. If you feel that an IND is not required, justify in the text box below how this study meets the criteria for 21CFR312.2.**

No, this research study does not involve a new use of an approved drug.

**26.6 List all FDA approved drugs being used in accordance with FDA labeling which will be administered as part of this research study. Explain how they will be stored and under whose supervision they will be administered.**

400 IU or 2000 IU capsules.

## 27.0 Device Studies

**27.1 If this study involves the use of a device, complete the following items as they apply.**

**Categorize the device:**

- [ ] FDA approved device. The FDA has approved this device for the indication and manner described in this study. (Attach FDA 510K or the device brochure to Section S.)
- [ ] Experimental use of an approved device. This may require an IDE.
- [ ] Humanitarian use device.
- [ ] Experimental device (Requires an IDE or NSRD determination).

---

**Study Devices:**

**Please list all devices involved in this study. If this study was migrated from INSPIR I, please re-list here all devices that were listed in the read only question shown above.**

No Device has been associated.

**Device Risk**

When research is conducted on non-approved devices or involves the experimental use of approved devices, the IRB must classify the device as "significant risk" or "non-significant risk". The risk determination of a device is based on the “proposed use” of the device, and not on the device alone.
If you believe this study involves an NSR (non-significant risk) device, please justify this determination in the text box below. *Attach any supporting documentation (summary reports of prior investigations with the device, documentation from an independent source verifying that the device operates within safety guidelines, etc.)*

**Does this study involve the use of an investigational electrical device?**

○ Yes ◉ No

For BUMC/BMC Investigators only: If yes, the device must be approved by Biomedical Engineering and this application will be routed there for approval signature. You must take your device to Biomedical Engineering for review.

**27.2 Study Attachments**

Here you can attach any study related documents including, but not limited to, device related documents.
*Please attach a copy of the investigator's device brochure for each device listed above (if any).*

No electronic document has been associated.

**27.3 Read Only. This question was imported from INSPIR I (if applicable) to help fill out the sections listed above.**

If applicable, provide the IDE number / HDE number. State the name of the device, the manufacturer and who holds the IDE. (A copy of the investigator's device brochure must be attached.)

## 28.0 Study Attachments

**28.1 Attach here any remaining study documents other than the ones listed below.**

No electronic document has been associated.
